# Expansion of the Strigolactone Profluorescent Probes Repertory: The Right Probe for the Right Application

**DOI:** 10.3389/fpls.2022.887347

**Published:** 2022-06-02

**Authors:** Alexandre de Saint Germain, Guillaume Clavé, Paul Schouveiler, Jean-Paul Pillot, Abhay-Veer Singh, Arnaud Chevalier, Suzanne Daignan Fornier, Ambre Guillory, Sandrine Bonhomme, Catherine Rameau, François-Didier Boyer

**Affiliations:** ^1^Université Paris-Saclay, INRAE, AgroParisTech, Institut Jean-Pierre Bourgin (IJPB), Versailles, France; ^2^Université Paris-Saclay, CNRS, Institut de Chimie des Substances Naturelles, Gif-sur-Yvette, France

**Keywords:** strigolactone, profluorescent probes, pea, *Arabidopsis thaliana*, *Physcomitrium patens*, α/β-hydrolases, plant hormone, structure-activity relationship

## Abstract

Strigolactones (SLs) are intriguing phytohormones that not only regulate plant development and architecture but also interact with other organisms in the rhizosphere as root parasitic plants (*Striga, Orobanche*, and *Phelipanche*) and arbuscular mycorrhizal fungi. Starting with a pioneering work in 2003 for the isolation and identification of the SL receptor in parasitic weeds, fluorescence labeling of analogs has proven a major strategy to gain knowledge in SL perception and signaling. Here, we present novel chemical tools for understanding the SL perception based on the enzymatic properties of SL receptors. We designed different profluorescent SL Guillaume Clavé (GC) probes and performed structure-activity relationship studies on pea, *Arabidopsis thaliana*, and *Physcomitrium* (formerly *Physcomitrella*) *patens*. The binding of the GC probes to PsD14/RMS3, AtD14, and OsD14 proteins was tested. We demonstrated that coumarin-based profluorescent probes were highly bioactive and well-adapted to dissect the enzymatic properties of SL receptors in pea and a resorufin profluorescent probe in moss, contrary to the commercially available fluorescein profluorescent probe, Yoshimulactone Green (YLG). These probes offer novel opportunities for the studies of SL in various plants.

## Introduction

Bioactive fluorescent-labeled plant hormones are highly valuable tools in hormone research either to address the mechanism of hormone transport and to obtain quantitative data on the dynamic of hormone levels or in the search for novel agonists or antagonists *via* the screening of chemical libraries (Lace and Prandi, 2016; Geisler, 2018; Balcerowicz et al., 2021). These probes are generally designed to retain the original hormonal activity and to activate signaling by binding to hormone receptors. For *in planta* imaging, the fluorophores should possess the best molecular brightness (Grimm and Lavis, 2022) and the detection of their fluorescence should not be affected by tissue autofluorescence (García-Plazaola et al., 2015). Indeed, the high abundance of endogenous fluorescent molecules (e.g., not only chlorophyll but also lignin, carotenes, xanthophylls, flavonoids, anthocyanins, alkaloids, etc.) is a real challenge for *in vivo* imaging in plants (Donaldson, 2020). For this purpose, the best spectral suitable window is reported to be between 550 and 650 nm for excitation and emission wavelengths of fluorophores. Due to these specific properties demanded in plant research, the available fluorophores are limited in this context (Grimm and Lavis, 2022).

Strigolactones are the last discovered class of phytohormones, controlling shoot branching, and many other aspects of plant development in vascular and non-vascular plants (Gomez-Roldan et al., 2008; Umehara et al., 2008; Proust et al., 2011; Lopez-Obando et al., 2015). They were first discovered as key signals in the rhizosphere as signaling the presence of a host root for parasitic plants and for arbuscular mycorrhizal fungi (AMF; Cook et al., 1966; Akiyama et al., 2005; Xie et al., 2010).

**Figure 1:**
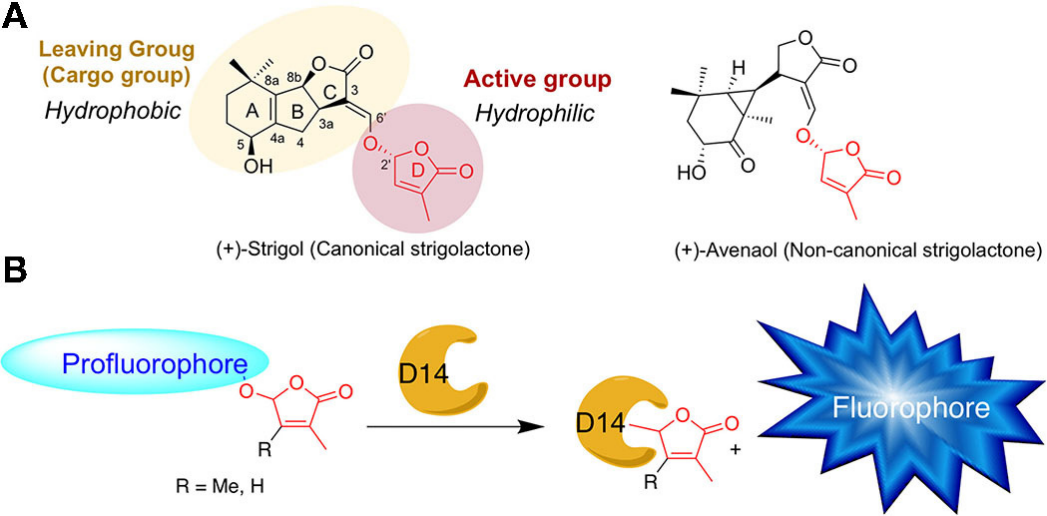
**(A)** Chemical structures of natural strigolactones. **(B)** Principle of SL profluorescent probes. D14 = SL receptor.

Strigolactones are a large family of specialized metabolite and to date, more than 30 natural SLs have been identified in root exudates of various plants (Yoneyama, 2020). SLs are derived from all-*trans*-β-carotene and are characterized by two specific chemical groups: an invariant butenolide D-ring bearing a 4′-methyl group and a structurally variable cargo group, linked by an enol ether bridge (Figure 1A). This connection has a 2'*R* configuration that is highly conserved in natural SLs (de Saint Germain et al., 2013; Yoneyama, 2020). SLs are classified into two distinct types: canonical SLs which have the cargo group containing an ABC tricycle and non-canonical SLs with the absence of the ABC tricycle (Figure 1A; Yoneyama et al., 2018). Based on the structure–activity relationship studies, it has been demonstrated that the D-ring is absolutely required for the SL bioactivity and can be qualified as an active group, whereas the cargo group can be drastically modified or even replaced by another hydrophobic group (i.e., in Debranone or Nijmegen; Takahashi and Asami, 2018).

In seed plants, SL perception as phytohormone involves a receptor called, DWARF14 (D14), [OsD14 in rice, AtD14 in Arabidopsis, RAMOSUS3 (RMS3) in pea, DECREASED APICAL DOMINANCE2 (DAD2) in petunia] which belongs to the α/β hydrolase family with a conserved catalytic triad (Ser, His, Asp; Arite et al., 2009; Hamiaux et al., 2012; Waters et al., 2012; de Saint Germain et al., 2016; Yao et al., 2016). In *Physcomitrium patens* and in obligate root parasitic plants, SLs are perceived by their ancestral paralogs, HYPOSENSITIVE TO LIGHT/KARRIKIN INSENSITIVE2 (HTL/KAI2) (Conn et al., 2015; Toh et al., 2015; de Saint Germain et al., 2021b; Lopez-Obando et al., 2021; Mizuno et al., 2021), referred hereinafter as KAI2s.

Interestingly, the D14 and KAI2 proteins can interact and cleave SLs, releasing the cargo group, which can therefore be called leaving group. To decipher the SL perception mechanism, bioactive fluorescent SL mimics were designed by different groups to investigate and characterize the mechanism of SL perception in multiple organisms (non-vascular and seed plants, including root parasitic plants and fungi). SL fluorescent probes have been developed since 2003 as tracers to investigate the spatiotemporal distribution of SLs in plants and fungi (Reizelman et al., 2003; Prandi et al., 2014; Lace and Prandi, 2016; Van Overtveldt et al., 2019). However, these fluorescence-based approaches allow no distinction between intact and hydrolyzed SL analogs, which may be an important drawback for data analyses.

Thanks to the structure–activity relationship (SAR) studies, fluorescent-labeled SLs have been designed by replacing the editable SL cargo group with a fluorophore, which becomes fluorescent only after perception and cleavage of the D-ring (Figure 1B). These so-called profluorescent probes allow the dynamic/temporal monitoring of the enzymatic activity of SL receptors *in vitro* (Tsuchiya et al., 2015; de Saint Germain et al., 2016; Wang et al., 2021) and *in planta* (Tsuchiya et al., 2015, 2018; Wang et al., 2021).

**Figure 2:**
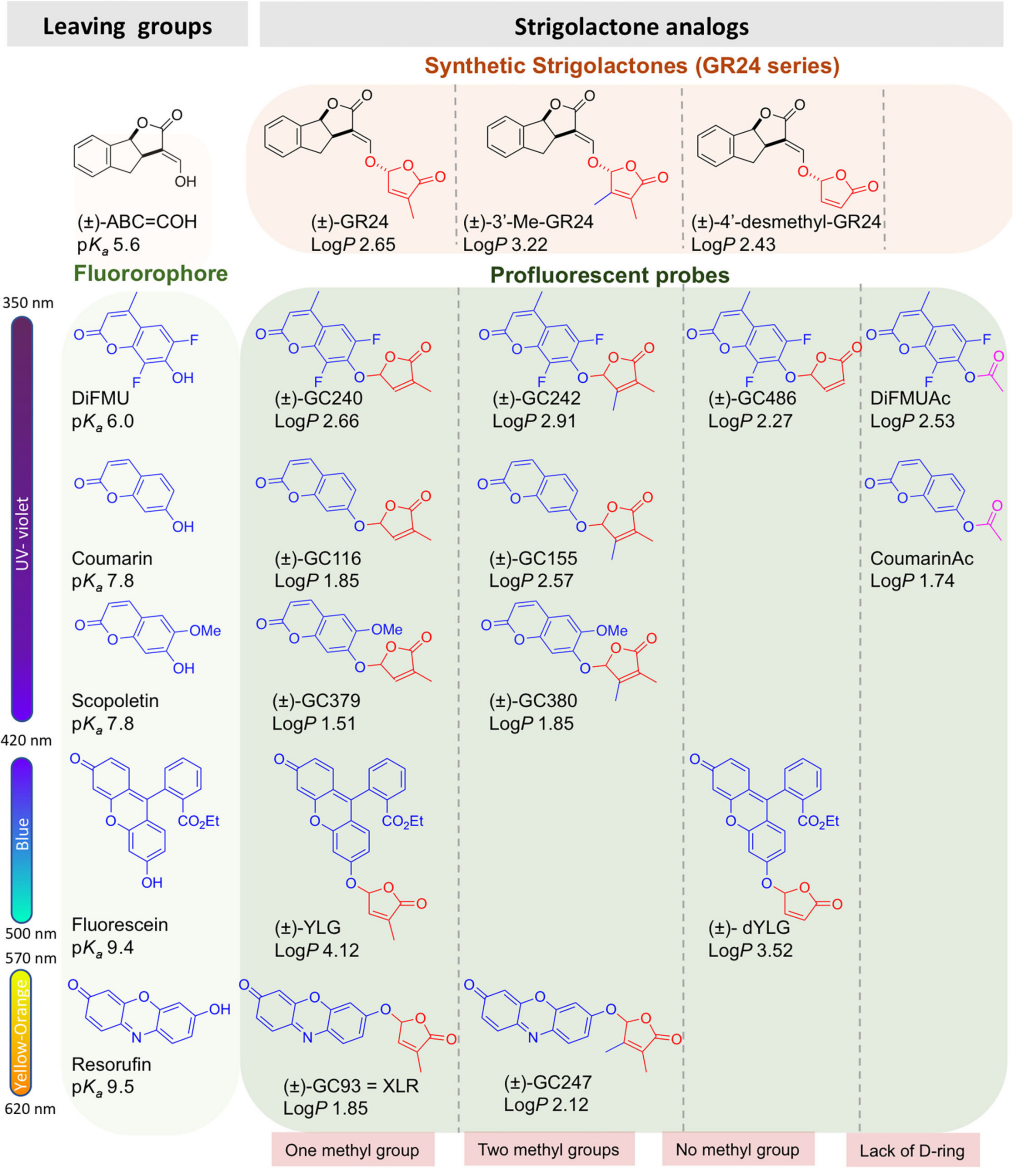
Chemical probes discussed in this study. Log*P* and p*K*_*a*_ are calculated by the ACD program.

The profluorescent probes include the Guillaume Clavé (GC) series, made of molecules bearing the 6,8-difluoro-7-hydroxy-4-methyl-2*H*-chromen-2-one (DiFMU) profluorescent moiety, either connected to a non-methylated [(±)-GC486] group, a mono-methylated [(±)-GC240] group, or a dimethylated [(±)-GC242] D-ring (de Saint Germain et al., 2016; Figures 1B, 2). *In vitro* enzymatic assays carried out with these probes revealed two-phase cleavage kinetics. The presence of a second phase with a plateau or a curve with a low slope suggests the formation of a relatively stable covalent adduct to the protein.

In the hypothetical model, the cleavage activity of SL receptors could be a way to stabilize the interaction between D14 and SL by a covalent link. From this model, an unusual hormonal perception mechanism has been proposed in which SLs are cleaved by the D14 receptor and form a covalent adduct linked to the histidine residue of the catalytic triad. Upon SL cleavage and perception, the D14 interacts with signaling partners to transduce the hormonal signal (de Saint Germain et al., 2016; Yao et al., 2016; Shabek et al., 2018). More recently, another SL perception mechanism independent of the enzymatic activity has been proposed (Seto et al., 2019). It highlights the necessity to develop innovative tools to better characterize the kinetics of SL perception (Bürger and Chory, 2020).

The GC series of profluorescent probes has also been recently used to characterize enzymatic properties of other putative SL receptors, such as PrKAI2d3 from *Phelipanche ramosa* root parasitic plant (de Saint Germain et al., 2021b) and PpKAI2L proteins from *Physcomitrium patens* (Lopez-Obando et al., 2021). Desmethyl profluorescent probes are particularly relevant for investigating the KAI2 pathway as the preference of this ancient pathway for desmethyl butenolides was recently demonstrated, and the role of (−)-desmethyl GR24 as an agonist of KAI2 protein was highlighted (Yao et al., 2021).

Yoshimulactone Green (YLG) is another profluorescent probe based on a fluorescein moiety linked to the D-ring by an ether bond (Figure 2; Tsuchiya et al., 2015). It has been developed for the characterization of SL receptors from *Striga* root parasitic plants, especially ShHTL7. The mechanism of SL perception by ShHTL7 was demonstrated to be similar to that of the D14 protein (Yao et al., 2017). The development of a variant of YLG (YLGW) allowed for the visualization of SL receptor activity in germinating *Striga hermonthica* seeds (Tsuchiya et al., 2018). The YLG is commercially available and has been used thereafter to identify SL receptor antagonists as tolfenamic acid (Hamiaux et al., 2018), KK094, and DL1b (Nakamura et al., 2019; Yoshimura et al., 2020) toward DAD2 and AtD14 proteins, respectively, highlighting the usefulness of this probe. Very recently, a novel resorufin-based SL profluorescent probe, Xilatone Red (XLR) based on a resorufin moiety has been developed (Wang et al., 2021).

Due to the structural diversities of SL receptors from different organisms, as well as the different functions of SL as a plant hormone and/or as a rhizospheric signal, the search for novel profluorescent probes is still necessary. For example, ShD14 is not able to cleave YLG whereas it could cleave (±)-GR24 (Xu et al., 2018). Here, we designed and characterized other different profluorescent SL mimic series with three different fluorophores (coumarin, scopoletin, and resorufin). These mimics have various physicochemical (Log*P*, p*K*_*a*_) and optical properties and bear a different number of methyl groups on the D-ring, aimed at meeting specific requirements for SL research. Their bioactivity for the control of shoot branching in pea, Arabidopsis, and for controlling moss development was evaluated. Their biochemical characterization was also performed with all four characterized SL receptors from flowering plants: AtD14, OsD14, DAD2, and RMS3.

## Materials and Methods

### Chemistry, General Experimental Procedure

All non-aqueous reactions were run under an inert atmosphere (argon), by using standard techniques for manipulating air-sensitive compounds. All glassware were stored in the oven and/or were flame-dried prior to use. Anhydrous solvents were obtained by filtration through drying columns. Analytical thin-layer chromatography (TLC) was performed on plates precoated with silica gel layers. Compounds were visualized by one or more of the following methods: (1) illumination with a short wavelength UV lamp (i.e., λ = 254 nm) and (2) spraying with a 3.5% (w/v) phosphomolybdic acid solution in absolute ethanol. Flash column chromatography was performed using 40–63 mesh silica. Nuclear magnetic resonance spectra (^1^H; ^13^C NMR) were recorded at [300; 75] MHz on a Bruker DPX 300 spectrometer. For the ^1^H spectra, data are reported as follows: chemical shift, multiplicity (s = singlet, d = doublet, t = triplet, q = quartet, m = multiplet, bs = broad singlet, coupling constant in Hz and integration). Infrared (IR) spectra are reported in reciprocal centimeters (cm^−1^). Buffers and aqueous mobile-phases for high-performance liquid chromatography (HPLC) were prepared using water purified with a Milli-Q system (purified to 18.2 MΩcm). Analytical ultra-performance liquid chromatography (UPLC) was performed on an Acquity Waters UPLC system equipped with a PDA and a mass spectrometer detector. Semi-preparative HPLC was performed on a Waters system equipped with 600 E pump system, a Waters 2,767 sample manager, injector and collector, and a waters PDA 2,996 UV-vis detector. Mass spectra (MS) and high-resolution mass spectra (HRMS) were determined by electrospray ionization (ESI) coupled to a time-of-flight analyzer (Waters LCT Premier XE). 7-Hydroxycoumarin (Coumarin) was synthesized according to the procedure of Timonen et al. (2011) in one step. 7-Acetoxycoumarin (CoumarinAc) was prepared according to the method of Confalone and Confalone (1980), and DiFMUAc was performed according to the method of Bürger et al. (2012). (±)-GC240, (±)-GC242, and (±)-GC486 were prepared according to the method of de Saint Germain et al. (2016); 5-bromo-4-methylfuran-2(5*H*)-one and 5-chloro-3,4-dimethylfuran-2(5H)-one were synthetized according to the procedure of Wolff and Hoffmann (1988) and Canévet and Graff (1978). (±)-GR24, (±)-ABC=CHOH tricycle [3-(hydroxymethylene)-3,3a,4,8b-tetrahydro-2*H*-indeno[1,2-*b*]furan-2-one] were prepared according to the method of Mangnus et al. (1992). (+)-GR24 was obtained as described by Lopez-Obando et al. (2021). DiFMU and (±)-YLG were purchased from Carbosynth™ and TCI™, respectively. All structures of GC probes were confirmed by NMR, IR, and HRMS analyses.

#### 7-[(4-Methyl-5-Oxo-2,5-Dihydrofuran-2-yl)Oxy]-4-Methyl-2H-1-Benzopyran-2-One [(±)-GC116]

To a solution of 5-bromo-3-methylfuran-2(5*H*)-one (448 mg, 3.10 mmol), 7-hydroxycoumarin (400 mg, 2.46 mmol), and *N, N*-diisopropylethylamine (DIEA; 1.05 mL, 6.00 mmol) were sequentially added to MeCN (10.0 ml). The resulting mixture was stirred at room temperature and after 10 min, a white solid precipitated. The reaction was allowed to proceed for 14 h, and then checked for completion by TLC (heptane/EtOAc 3:2 v/v). A large part of the product was recovered by filtration and the remaining part was purified on a silica gel column (heptane/EtOAc 3:2 v/v) giving (±)-GC116 as a white solid (446 mg, 1.73 mmol, 70%). Mp 216°C. ^1^H-NMR (300 MHz, CDCl_3_) δ: 2.04 (s, 3H), 6.33–6.36 (m, 2H), 7.02–7.09 (m, 3H), 7.44–7.46 (d, *J* = 8.6 Hz, 1H), 7.65–7.67 (d, *J* = 9.8 Hz, 1H). ^13^C-NMR (125 MHz, CDCl_3_) δ: 10.5, 98.1, 104.7, 113.5, 114.6, 114.7, 129.2, 134.8, 141.8, 143.0, 155.3, 159.0, 160.6, 170.8. IR ν_max_ (film): 680, 746, 794, 841, 879, 956, 1,018, 1,092, 1,138, 1,165, 1,208, 1,283, 1,363, 1,508, 1,562, 1,622, 1,664, 1,730, 1,778, 3,078 cm^−1^. HRMS (ESI): *m/z* calc. for C_14_H_11_O_5_ [M + H]^+^: 259.0606, found: 259.0605.

#### 7-[(3,4-Dimethyl-5-Oxo-2,5-Dihydrofuran-2-yl)Oxy]-4-Methyl-2H-1-Benzopyran-2-One [(±)-GC155]

To a solution of 5-chloro-3,4-dimethylfuran-2(5*H*)-one (352 mg, 2.00 mmol; Canévet and Graff, 1978), 7-hydroxycoumarin (300 mg, 1.85 mmol), and DIEA (697 μL, 4.00 mmol) were sequentially added to MeCN (10 ml). The resulting mixture was stirred at room temperature for 14 h and then checked for completion by TLC (heptane/EtOAc 1:1 v/v). The crude was evaporated to dryness and then purified on a silica gel column (heptane/EtOAc 6:4 v/v) giving (±)-GC155 as a white solid (423 mg, 1.55 mmol, 84%). Mp 176°C. ^1^H-NMR (300 MHz, CDCl_3_) δ: 1.85 (t, *J* = 1.2 Hz, 3H), 2.04 (t, *J* = 0.9 Hz, 3H), 6.08 (s, 1H), 6.24–6.27 (d, *J* = 9.5 Hz, 1H), 6.98–7.01 (m, 2H), 7.37–7.39 (d, *J* = 8.1 Hz, 1H), 7.58–7.61 (d, *J* = 9.6 Hz, 1H). ^13^C-NMR (75 MHz, CDCl_3_) δ: 8.7, 11.7, 99.9, 104.6, 113.5, 114.8, 114.9, 127.5, 129.3, 143.1, 153.3, 155.5, 159.5, 160.7, 171.4. IR ν_max_ (film): 674, 661, 750, 834, 886, 975, 1,052, 1,088, 1,131, 1,162, 1,195, 1,236, 1,285, 1,318, 1,361, 1,387, 1,505, 1,565, 1,615, 1,624, 1,689, 1,745, 1,781, 3,081 cm^−1^. HRMS (ESI): *m/z* calc. for C_15_H_13_O_5_ [M + H]^+^: 273.0718, found: 273.0753.

#### 7-[(4-Methyl-5-Oxo-2,5-Dihydrofuran-2-yl)Oxy]-6-Methoxy-4-Methyl-2H-1-Benzopyran-2-One [(±)-GC379]

To a solution of 5-bromo-3-methylfuran-2(5*H*)-one (53.0 mg, 300 μmol), scopoletin (30.0 mg, 156 μmol) and DIEA (156 μmol, 900 μmol) were sequentially added to MeCN (4 mL) The resulting mixture was stirred at room temperature for 14 h, and then checked for completion by TLC (heptane/EtOAc 1:1 v/v). The crude was evaporated to dryness and then purified on a silica gel column (heptane/EtOAc 1:1 v/v) giving (±)-GC379 as a white solid (43.0 mg, 149 μmol, 96%). Mp 164°C. ^1^H-NMR (300 MHz, CDCl_3_) δ: 2.01 (s, 3H), 3.90 (s, 3H), 6.33-6.36 (m, 2H), 6.92 (s, 1H), 7.06–7.07 (t, *J* = 1.6 Hz, 1H), 7.21 (s, 1H), 7.61–7.63 (d, *J* = 9.5 Hz, 1H). ^13^C-NMR (125 MHz, CDCl_3_) δ: 10.8, 56.5, 98.8, 106.1, 109.2, 114.4, 115.4, 135.2, 141.9, 142.9, 147.0, 148.6, 149.1, 160.9, 170.9. IR ν_max_ (film): 820, 869, 928, 954, 1,014, 1,072, 1,099, 1,147, 1,173, 1,196, 1,214, 1,250, 1,278, 1,376, 1,390, 1,423, 1,459, 1,512, 1,568, 1,616, 1,721, 1,776 cm^−1^. HRMS (ESI): *m/z* calc. for C_15_H_13_O_6_ [M + H]^+^: 289.0712, found: 289.0714.

#### 7-[(3,4-Dimethyl-5-Oxo-2,5-Dihydrofuran-2-yl)Oxy]-6-Methoxy-4-Methyl-2H-1-Benzopyran-2-One [(±)-GC380]

To a solution of 5-chloro-3,4-dimethylfuran-2(5*H*)-one (43.0 mg, 300 μmol), scopoletin (30.0 mg, 156 μmol) and DIEA (156 μmol, 900 μmol) were sequentially to MeCN (4 mL) added. The resulting mixture was stirred at room temperature for 14 h, and then checked for completion by TLC (heptane/EtOAc 1:1 v/v). The crude was evaporated to dryness and then purified on a silica gel column (heptane/EtOAc 1:1 v/v) giving (±)-GC380 as a white solid (32.0 mg, 106 μmol, 68%). Mp 172°C. ^1^H-NMR (300 MHz, CDCl_3_) δ: 1.88–1.89 (t, *J* = 1.5 Hz, 3H), 2.12–0.13 (t, *J* = 0.9 Hz, 3H), 3.89 (s, 3H), 6.12 (s, 1H), 6.31–6.34 (d, *J* = 9.5 Hz, 2H), 6.92 (s, 1H), 7.20 (s, 1H), 7.61–7.64 (d, *J* = 9.6 Hz, 1H). ^13^C-NMR (75 MHz, CDCl_3_) δ: 8.7, 11.8, 56.5, 100.8, 106.4, 109.3, 114.4, 115.4, 127.5, 143.0, 147.2, 149.0, 149.1, 153.4, 160.9, 171.5. IR ν_max_ (film): 750, 817, 850, 860, 922, 972, 1,016, 1,053, 1,096, 1,143, 1,172, 1,194, 1,246, 1,276, 1,369, 1,387, 1,423, 1,513, 1,568, 1,615, 1,720, 1,776, 2,851, 2,924, 3,065 cm^−1^. HRMS (ESI): *m/z* calc. for C_16_H_15_O_6_ [M + H]^+^: 303.0869, found: 303.0872.

#### 7-[(4-Methyl-5-Oxo-2,5-Dihydrofuran-2-yl)Oxy]**-3H-Phenoxazin-3-One** [(±)-GC93]

To a solution of 5-bromo-3-methylfuran-2(5*H*)-one (51.0 mg, 290 μmol), resorufin sodium salt (65.0 mg, 277 μmol) and DIEA (1.05 ml, 6.00 mmol) were sequentially added to DMF (4 ml). The resulting mixture was stirred at room temperature for 14 h and then checked for completion by TLC (CH_2_Cl_2_/EtOAc 8:2 v/v). The crude was diluted with EtOAc, successively washed with 10% aqueous citric acid, brine, dried over Na_2_SO_4_, and evaporated to dryness. The resulting residue was purified by chromatography on a silica gel column with a step gradient of EtOAc (0-10% v/v) in CH_2_Cl_2_ as the mobile phase, giving (±)-GC93 as yellow solid (53.0 mg, 172 μmol, 62%). Mp decomposition at 244°C. ^1^H-NMR (300 MHz, CDCl_3_) δ: 2.06–2.07 (t, *J* = 1.6 Hz, 3H), 6.33–6.34 (d, *J* = 2.1 Hz, 1H), 6.38–6.39 (t, *J* = 1.6 Hz, 1H), 7.44–7.46 (dd, *J*_1_ = 9.9 Hz, *J*_2_ = 2.0 Hz, 1H), 7.04–7.05 (t, *J* = 1.7 Hz, 1H), 7.10–7.14 (m, 2H), 7.41–7.44 (d, *J* = 9.9 Hz, 1H), 7.75–7.78 (d, *J* = 8.3 Hz, 1H). RMN ^13^C (75 MHz, CDCl_3_) δ: 10.9, 98.1, 103.8, 107.3, 114.7, 129.9, 131.9, 134.9, 135.2, 141.6, 145.3, 145.4, 147.3, 149.6, 159.6, 170.3, 186.5. IR ν_max_ (film): 711, 742, 758, 782, 799, 817, 831, 862, 907, 950, 976, 994, 1,016, 1,036, 1,078, 1,096, 1,159, 1,210, 1,251, 1,319, 1,336, 1,366, 1,448, 1,480, 1,505, 1,561, 1,590, 1,642, 1,775, 2,926, 3,043, 3,094 cm^−1^. HRMS (ESI): *m/z* calc. for C_17_H_12_NO_5_ [M + H]^+^: 310.0715, found: 310.0766.

#### 7-[(3,4-Dimethyl-5-Oxo-2,5-Dihydrofuran-2-yl)Oxy]**-3H-Phenoxazin-3-One** [(±)-GC247]

To a solution of 5-chloro-3,4-dimethylfuran-2(5*H*)-one (35.0 mg, 240 μmol), resorufin sodium salt (28.2 mg, 120 μmol) and DIEA [84.0 μL, 480 μmol were sequentially added to DMF (2 ml)]. The resulting mixture was stirred at 64°C for 14 h. and then checked for completion by TLC (CH_2_Cl_2_/EtOAc 8:2). The crude mixture was diluted with EtOAc, successively washed with 10% aqueous citric acid, brine, dried over Na_2_SO_4_, and evaporated to dryness. The resulting residue was purified by chromatography on a silica gel column with a step gradient of EtOAc (0–20% v/v) in CH_2_Cl_2_ as the mobile phase, giving (±)-GC247 as yellow solid (25.0 mg, 77.0 μmol, 64%). Mp decomposition at 246°C. ^1^H-NMR (300 MHz, CDCl_3_) δ: 1.92–1.93 (t, *J* = 1.5 Hz, 3H), 2.11–2.12 (t, *J* = 1.6 Hz, 3H), 6.18 (bs, 1H), 6.32–6.33 (d, *J* = 2 Hz, 1H), 6.82–6.86 (dd, *J*_1_ = 9.8 Hz, *J*_2_ = 2.1 Hz, 1H), 7.04–7.05 (t, *J* = 1.7 Hz, 1H), 7.10–7.15 (m, 1H), 7.40–7.44 (d, *J* = 9.9 Hz, 1H), 7.74–7.77 (dd, *J*_1_ = 9.8 Hz, *J*_2_ = 0.6 Hz, 1H). ^13^C-NMR (75 MHz, CDCl_3_) δ: 8.7, 11.8, 99.7, 103.7, 107.2, 114.7, 127.6, 129.8, 131.8, 134.8, 134.9, 145.3, 147.1, 149.6, 153.1, 159.9, 171.3, 186.4. IR ν_max_ (film): 711, 742, 758, 782, 799, 817, 831, 862, 907, 950, 976, 994, 1,016, 1,036, 1,078, 1,096, 1,159, 1,210, 1,251, 1,319, 1,336, 1,366, 1,448, 1,480, 1,505, 1,561, 1,590, 1,642, 1,775, 2,926, 3,043, 3,094 cm^−1^. HRMS (ESI): *m/z* calc. for C_18_H_14_NO_5_ [M + H]^+^: 324.0872, found: 324.0857.

#### Stability of CoumarinAc and DiFMUAc in Dimethyl Sulfoxide

Dimethyl sulfoxide **(**DMSO) solution of the compound to be tested (1 mM) was incubated at 20°C in the HPLC vials. (±)-1-Indanol [Alfa Aesar, purity >97.5% (GC); 10 mM] was used as the internal standard. The samples were subjected to reverse-phase-ultra-performance liquid chromatography (RP-UPLC)-MS analyses by means of UPLC system equipped with a photo diode array (PDA) and a triple quadrupole detector (TQD) mass spectrometer (Acquity UPLC-TQD, Waters). RP-UPLC (HSS C_18_ column, 1.8 μm, 2.1 × 50 mm) with 0.1% (v/v) of formic acid in CH_3_CN and 0.1% (v/v) of formic acid in water (aq. FA, 0.1%, v/v, pH 2.8) were used as eluents [10% CH_3_CN, followed by linear gradient from 10 to 100% of CH_3_CN (4 min)] at a flow rate of 0.6 ml min^−1^. The detection was done by PDA and with the TQD mass spectrometer operated in electrospray ionization-positive mode at 3.2 kV capillary voltage. To maximize the signal, the cone voltage and collision energy were optimized to 20 V and 12 eV, respectively. The collision gas used was argon at a pressure maintained near 4.5 10^−3^ mBar. The relative quantity of the remaining (non-degraded) product was determined by integration comparison with the internal standard.

### Expression and Purification of Proteins

Expression and purification of RMS3, AtD14, DAD2, and OsD14 proteins with cleavable GST tag were performed in accordance with the study by de Saint Germain et al. (2016) and de Saint Germain et al. (2021b). For DAD2 protein expression, the full-length coding sequences from *Petunia hybrida* were amplified by PCR using cDNA as template and specific primers (DAD2_attb1_HRV3C (5′-ggggacaagtttgtacaaaaaagcaggctccctg gaagtgctgtttcagggcccg**ATG**G GACAGACCCTTTTAGA-3′) and DAD2_attb2 (5′-ggggaccactttgtacaagaaagct gggtc**tcaTCA**CCTATGTGA AAGAGCTCTTC-3′) containing a protease cleavage site for tag removal, and subsequently cloned into the pGEXT-4T-3 expression vector. Similarly, for OsD14 protein expression, the coding sequences from *Ozyza sativa* were deleted from 153 nucleotides (51 amino acid) amplified by PCR using cDNA as template and specific primers (OsD14Δ51_attb1_HRV3C (5′-ggggacaagtttgtacaaaaaagcag gctccctggaagtgctgtttcagggcccg **ATG**CCGAGCGGGGCGAAGCTGCTGC-3′) and OsD14Δ51_attb2 (5′-ggggaccact ttgtacaagaaagctgggtc**tcaTTA** GTACCGGGCGAGAGCGCGGCGGAG-3′).

### Method for Log*P* and p*K_*a*_* Calculation

Relative hydrophobicity (log*P*) and p*K*_*a*_ values of SL probes and fluorophores were calculated using the ACD program (Advanced Chemistry Development, Inc.: https://ilab.acdlabs.com/ilab2/).

### Pea Shoot Branching Assay

Pea (*Pisum sativum*) branching mutant plants used in this study were described previously (Rameau et al., 1997). The SL biosynthesis *rms1-10* (M3T-884) and SL response *rms3-4* (M2T-30) mutants were obtained from the dwarf cv. Térèse. Plants were grown in a greenhouse under long days as described by Braun et al. (2012).

#### Pea Shoot-Branching Assay by Direct Application on the Bud

The compounds to be tested were applied directly to the axillary bud with a micropipette as 10 μL of a solution containing 0.1% of acetone with 2% of polyethylene glycol 1,450, 50% of ethanol, and 0.4% of DMSO. The control 0 is the treatment with 0.1% of DMSO without compound. A total of 24 plants were sown per treatment in trays (2 repetitions of 12 plants). The treatment was done 8 days after sowing, on the axillary bud at node 3. The branches at nodes 1–2 were removed to encourage the outgrowth of axillary buds at nodes above. Nodes were numbered acropetally from the first scale leaf as node 1 and cotyledonary node as node 0. Bud growth at node 3 was measured 10 days after treatment. Plants with damaged main shoot apex or showing a dead white treated bud were discarded from the analysis. The SL-deficient *rms1-10* pea mutant was used for all experiments and WT Térèse as control. SL-perceived *rms3-4* pea mutant was used to test that when bioactive, the analog acts *via* RMS3, and it was also used to check the putative toxicity of probes.

#### Pea Shoot-Branching Assay by Vascular Supply

The compounds to be tested were applied by vascular supply (Muñoz et al., 2021). The control was the treatment with 0.1% of DMSO in water. A total of 12 plants were sown per treatment in trays and were treated with probes under node 3 bud generally 10 days after sowing. Compounds in DMSO solution were diluted with water to 3,000 nM for a treatment with 0.1% (v/v) DMSO. The branches at nodes 1 and 2 were removed to encourage the outgrowth of axillary buds at the nodes above. Nodes were numbered acropetally from the first scale leaf as node 1 and cotyledonary node as node 0. Bud growth at nodes 3 and 4 was measured with digital calipers 8–10 days after treatment. Plants with damaged main shoot apex or with a dead white treated-bud were discarded from the analysis. The SL-deficient *rms1-10* pea mutant was used for all experiments.

### Hydroponic Assay on Arabidopsis

The hydroponic assay was adapted from the study by Cornet et al. (2021). Seeds were surface-sterilized for 8 min in a solution of ethanol (95%) and hypochlorite solution (10%; Bayrol, Mundolsheim, France) and were rinsed two times with ethanol (100%). Each seed was sown on top of a cut 0.5 ml Eppendorf tube filled with agar medium containing 0.65% of agar and 10% of nutritive solution of 5 mM NO_3_. Tubes were soaked in water and stored in the dark at 4°C for 2 days. Twelve plants per pipette tip box (13 × 9 × 7 cm) were grown and supplied with nutrient solution as in the study by Boyer et al. (2014) at a concentration of 5 ml/L (750 ml of solution per box). Every week, the nutrient solution was renewed and every 10 days one time, a fresh batch of treatment was added to the solution. The first treatment occurred at day 27 after sowing when plants started to bolt. The number of rosette branches was counted at day 42.

### *Physcomitrium patens* Bioassay

Assays on *Physcomitrium patens* were performed on plants grown in 24-well plates, starting from very small pieces of moss tissues as described by Guillory and Bonhomme (2021). As for pea *rms1*, the *Ppccd8* SL synthesis mutant was used for assays, since the effect of the compounds was better seen in this mutant vs. wild type (WT; Lopez-Obando et al., 2021). For each treatment, 24 plants were grown in PpNO_3_ medium [minimal medium described by Ashton et al., 1979], dispatched in three different plates. Plants were grown for 2 weeks under control conditions, then treated with fluorophores or probes (all compounds used at 1 μM), before being placed vertically in the dark for 10 days. A single picture of each well was taken under an Axio Zoom microscope (Zeiss) with a dedicated program. Filaments were counted using ImageJ software (http://imagej.nih.gov/ij/) as described by Guillory and Bonhomme (2021). Twenty-four plants were tested in each treatment.

### Enzymatic Assays With Profluorescent Probes

The enzyme activity was determined by measuring the release of the fluorescent intensities of each fluorophores resulting from the cleavage of profluorescent probes by RMS3, AtD14, OsD14, and DAD2 proteins in a SPARK M10 in a 96-well format (de Saint Germain et al., 2021a). In the assay, using an Integra Viaflo 96 robot, 50 μL of a solution of protein at 0.33 μM in same buffer was added simultaneously in all 96 wells to 50 μL of profluorescent substrate solution (at varying concentrations, prepared from a 10 mM stock solution in 100% of DMSO) in PBS (100 mM of phosphate, pH 6.8, 150 mM of NaCl). After a lag time of 15 s, the formation of fluorophores was recorded over 3 h at 15 s intervals at 25°C. Each fluorophore was analyzed with the following excitation (ex) and emission (em) wavelengths: DiFMU λ_ex_ 360 nm/λ_em_ 450 nm, coumarin λ_ex_ 360 nm/λ_em_ 450 nm, resorufin λ_em_ 540/λ_ex_ 590 nm, and fluorescein λ_em_ 475 nm/λ_ex_ 520 nm. All experiments were repeated with three technical replicates. The fluorescence of each fluorophore was also determined for each measurement at the same time frame but in the absence of enzyme in order to determine the standard curves. For rapid enzymatic assays (**Figure 8A**, small panel), the solution of protein was added by the injector of the plate reader and then, the well was immediately read over 5 min with 1 s intervals. Same parameters were used to determine the fluorophore concentration.

### Statistical Analysis

Since deviations from normality were observed for axillary bud length after SL treatment in pea bioassay, the Kruskal–Wallis test was used to assess the significance of one treatment with one compound in comparison to treatment with another using R Commander version 1.7–3 (Fox, 2005). For the bioassay in moss, ANOVA and Tukey's test as *post-hoc* test was used.

## Results

### Design and Synthesis of SL Profluorescent Probes

#### SL Profluorescent Probes With Various Optical/Spectra Properties

**Figure 3:**
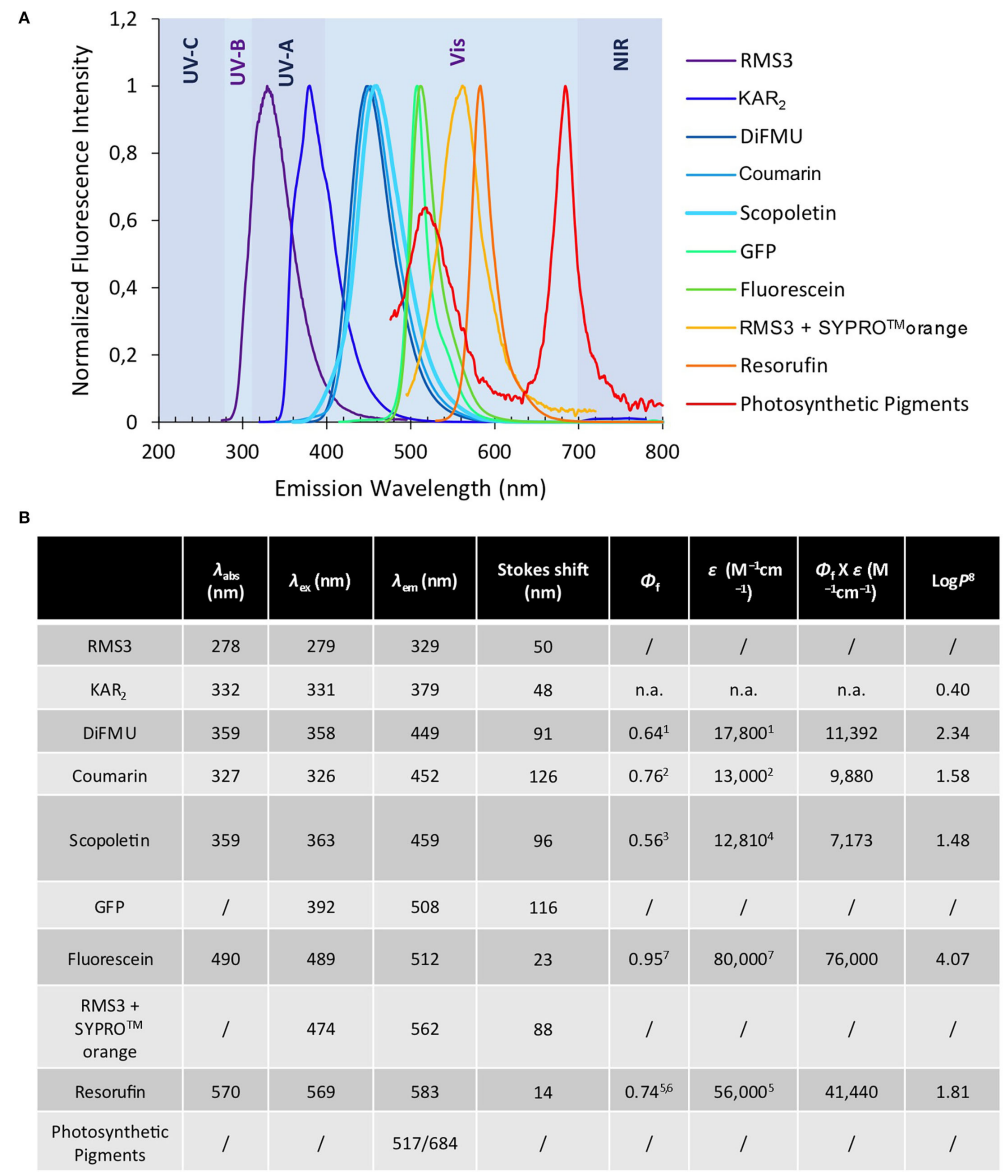
Optical properties of fluorophores. Normalized fluorescence emission spectra of the fluorophores and some chemical compounds **(A)** (mentioned in this study). Chemical and spectral data for each molecule as λ_abs_ (nm) λ_ex_ (nm) λ_em_ (nm) ε (M^−1^cm^−1^) Φ_f_ Φ_f_ X ε (“molecular brightness”) (M^−1^cm^−1^) Log*P* **(B)** ^1^pH 10 (Sun et al., 1998). ^2^pH 7.4 (Setsukinai et al., 2000). ^3^pH 6.8 (Pham et al., 2019). ^4^In EtOH (Abu-Eittah and El-Tawil, 1985). ^5^pH 9.5 (Tan et al., 2021). ^6^(Bueno et al., 2002). ^7^In 0.1 N NaOH, https://www.aatbio.com. ^8^Log*P* are calculated by the ACD program. n.a. not available.

We previously developed bioactive fluorogenic SL mimics, the racemic GC series, with commercially available coumarin moiety: 6,8-difluoro-7-hydroxy-4-methyl-2*H*-chromen-2-one (DiFMU) (±)-GC486, (±)-GC240, and (±)-GC242, respectively, with no, one, or two methyl groups on the bioactive group (de Saint Germain et al., 2016; Figure 2). For biochemical applications, the ideal fluorophore should exhibit a high molecular brightness (ε × Φ_f_, with ε as the extinction coefficient and Φ_f_ as the quantum yield), which considers the efficiencies of fluorescence and light absorption. The ideal fluorophore should possess a large difference between λ_ex_ and λ_em_ (called Stokes shift), no toxicity, a good aqueous solubility, good cell permeability, high stability, and a resistance to photobleaching. Among the fluorophores compatible with the definition of SL mimics, the DiFMU showed all these requirements, especially the better spectral properties: Stokes shift 97 nm and ε × Φ_f_ 17,800 M^−1^cm^−1^ (Figure 3, Supplementary Figure 1). Moreover, DiFMU was compatible with differential scanning fluorimetry (DSF) and intrinsic fluorescence assays since its emission spectrum does not overlap with those of protein dyed with SYPRO^TM^ orange and the intrinsic protein fluorescence (Figure 3).

To expand the repertory of SL profluorescent probes, new mimics have been designed with other fluorophores, such as coumarin [(±)-GC116, (±)-GC155] and scopoletin [(±)-GC379, (±)-GC380], bearing a methoxy group at the C-6 position and connected to D-ring butenolide with one or two methyl groups (Figure 2). These molecules could be valuable tools to study the effect of substitutions on the coumarin moiety, especially to evaluate the influence of the molecule reactivity (p*K*_*a*_ of the leaving group) and hydrophobicity (log*P*) on both biological and biochemical activity toward the various SL receptors, in order to perform SAR studies (Figure 2). A resorufin moiety was also targeted [(±)-GC93 = XLR (Wang et al., 2021), (±)-GC247 (Figure 2)], which has optical properties compatible for *in planta* imaging, contrary to coumarins. The excitation and emission maxima of resorufin (568 and 581 nm) and fluorescein (475 and 520 nm) made it suitable for use in plant tissue imaging compared to the other fluorophores (coumarin and DiFMU, 350–360 and 450–460 nm; Figure 3, Supplementary Figure 1). Likewise, resorufin allows for competitive enzymatic assay with UV fluorescent molecules like karrikins, for which intrinsic fluorescence assays are not possible.

#### SL Profluorescent Probes With Substitute D-Ring

To characterize the enzymatic properties of α/β hydrolase proteins like SL receptors, *para*-nitrophenyl acetate (*p-*NPA) is commonly used. Quantification of *p-*NPA hydrolysis is based on the measurement of absorbance, which has the disadvantage of requiring a large amount of protein in comparison to fluorescence-based detection. To overcome this drawback, we designed two fluorescent acetate probes (DiFMUAc and CoumarinAc) by acetylation of their phenolic moieties (Figure 2). These compounds could allow for the comparison of the enzymatic profile between probes and *per se* reveal the biological role of the D-ring.

#### Synthesis of SL Profluorescent Probes

**Figure 4:**
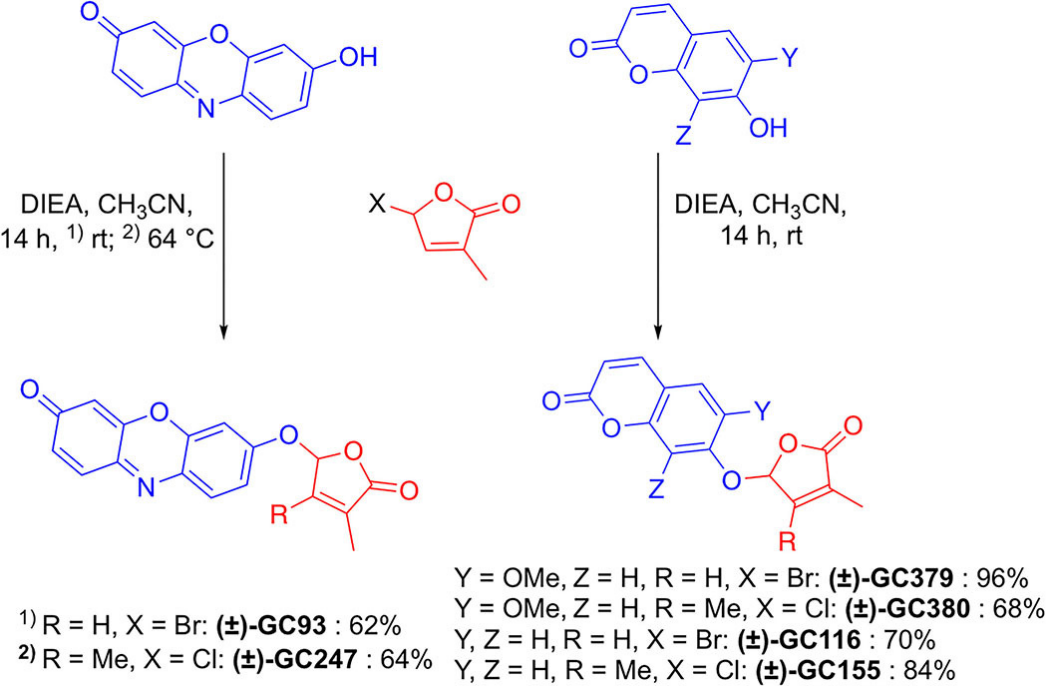
Synthesis of novel profluorescent SL probes.

GC probes have been prepared by the reaction of coumarins and resorufin with 5-bromo-3-methylfuran-2(5*H*)-one and 5-chloro-3,4-dimethylfuran-2(5*H*)-one and *N, N*-diisopropylethylamine as a base, in acetonitrile in yield up to 96% (Figure 4).

### Biological Activity of the Profluorescent Probes

#### Various Coumarin SL Profluorescent Probes Are Bioactive in Pea

**Figure 5:**
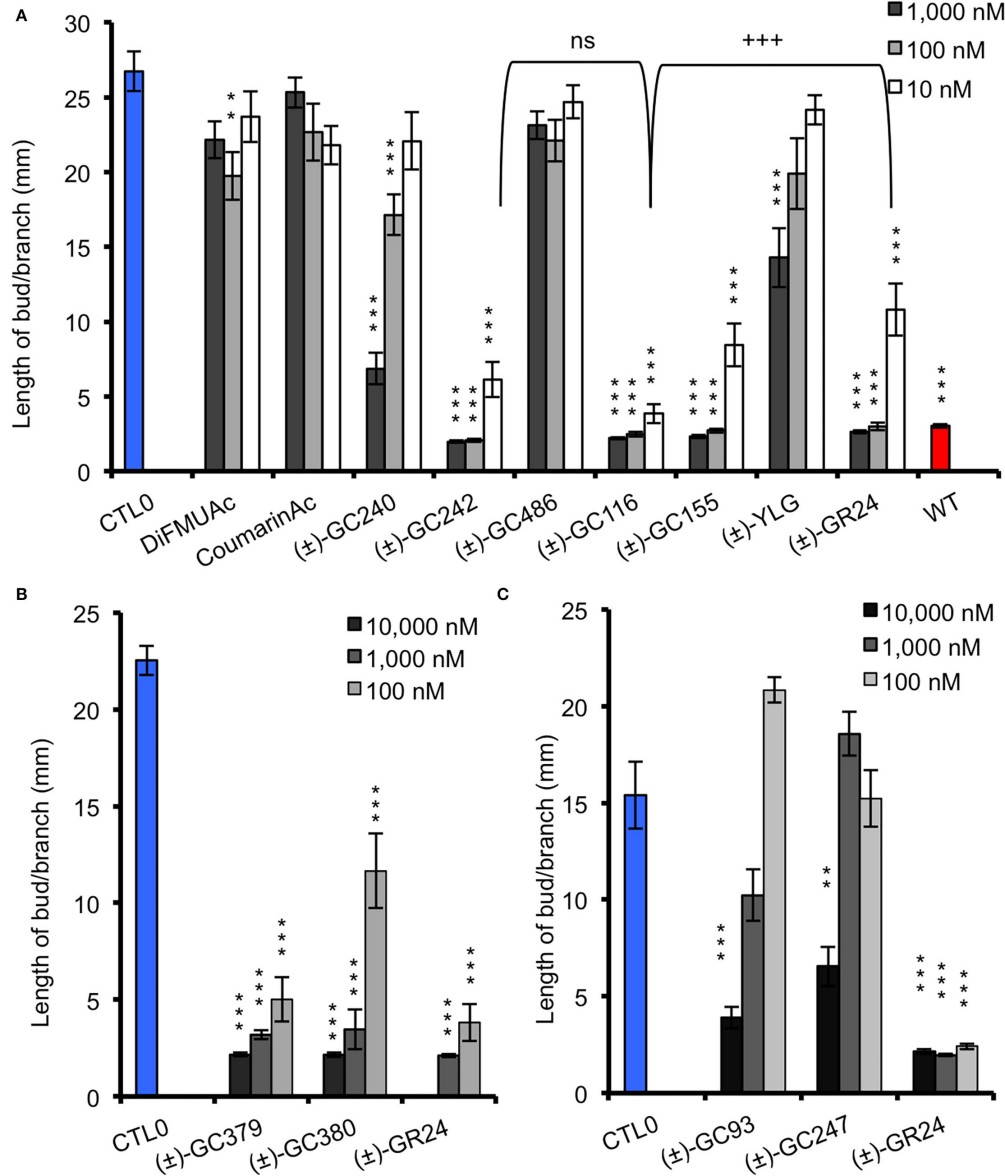
Bioactivity in pea of probes vs. (±)-GR24 (*rms1-10*), coumarin and fluorescein probes **(A)**, scopoletin probes **(B)**, and resorufin derivative probes **(C)**. Length of the axillary buds of *rms1-10* plants, 8 days after direct application of probes and of (±)-GR24. All replicates are presented in Supplementary Table 1 with control 0. These data were obtained from means ± SE (*n* ≥ 20 plants). **P < 0.01, ***P < 0.001 indicate significant differences with the control treatment (0 nM) (CTL0) (Kruskal–Wallis rank sum test). CTL0, control 0. All the replicates are presented in Supplementary Table 1. ^+++^*P* < 0.001 indicates significant differences with the (±)-GR24 treatment (10 nM) (Kruskal–Wallis rank sum test). ns, not significant.

In order to check whether the designed probes were biologically active on shoot branching inhibition, we performed branching assay with the SL-deficient *rms1-10* mutant of pea. If the probe is biologically active, it should inhibit branch development. To evaluate this inhibition, we compared our results with *rms1-10* mutants treated with a control solution, and with non-treated (NT) Térèse plants, for which bud development was inhibited by endogenous natural SLs. Globally, the acetate probes (DiFMUAc and CoumarinAc) showed no effect on *rms1-10* mutant plants, confirming that the D-ring group is essential for a significant biological effect (Figure 5A, Supplementary Table 1). The significant effect observed for DiFMUAc at 100 nM could be due to the slight toxicity of DIFMUAc on axillary bud; however, it is not detected at higher concentration.

Both (±)-GC242 and (±)-GC155 probes with two methyl groups on their D-ring appeared to be among the most bioactive molecules. We also observed an inhibition of bud development for the probes with one methyl group [(±)-GC240, (±)-GC116 and (±)-YLG] though the (±)-GC240 and (±)-YLG probes were less efficient than (±)-GR24 and probes with two methyl groups. This suggests that a two-methyl D-ring group improves the biological activity in pea as observed for SL analogs (Boyer et al., 2012, 2014). Surprisingly, when comparing the probes with one methyl group on the D-ring [(±)-GR24, (±)-GC116 and (±)-GC240], we observed the strongest inhibition of bud development, at 10 nM for (±)-GC116, suggesting that the coumarin moiety improved biological efficiency. Bearing scopoletin moiety (±)-GC379 and (±)-GC380 were bioactive for the three tested concentrations (Figure 5B). In contrast, the probes of the resorufin series [(±)-GC93 and (±)-GC247] showed an inhibitory effect with statistical significance only at 10 μM (Figure 5C). The (±)-YLG probe was less bioactive than (±)-GC240 and (±)-GC116 probes suggesting that the fluorescein group affected probe activity. We confirmed that (±)-GC486, without methyl on the D-ring, showed no biological activity on branching inhibition (de Saint Germain et al., 2016; Figure 5A) similar to (±)-dYLG (Yao et al., 2021). GC analogs could not repress branching of the pea *rms3-4* perception mutant (Supplementary Table 2). These results suggest that GC probes, such as (±)-GR24, are bioactive SL analogs and inhibit bud outgrowth in pea *via* the RMS3 receptor, and not because of toxicity.

In order to explain the lower bioactivity of resorufin probes [(±)-GC93, (±)-GC247] and fluorescein (±)-YLG (Figures 5A,C), we fed the SL analogs to the vascular stream of pea shoots as previously described (de Saint Germain et al., 2021b; Muñoz et al., 2021). This feeding method allowed to circumvent a putative problem of tissue penetration due to compound hydrophobicity; however, this is not highlighted by Log*P* modeling (partition coefficient; Figure 2). Again, we found lower bioactivity for (±)-YLG and (±)-GC93 compared to the coumarin derivatives series, ruling out the role of tissue penetration on the weak bioactivity (Supplementary Figure 2, Supplementary Table 3). If not hydrophobicity, the most plausible explanation could be relatively the bigger size of fluorescein and resorufin compared to coumarin moiety.

#### Bioactivity of GC Probes in Arabidopsis

**Figure 6:**
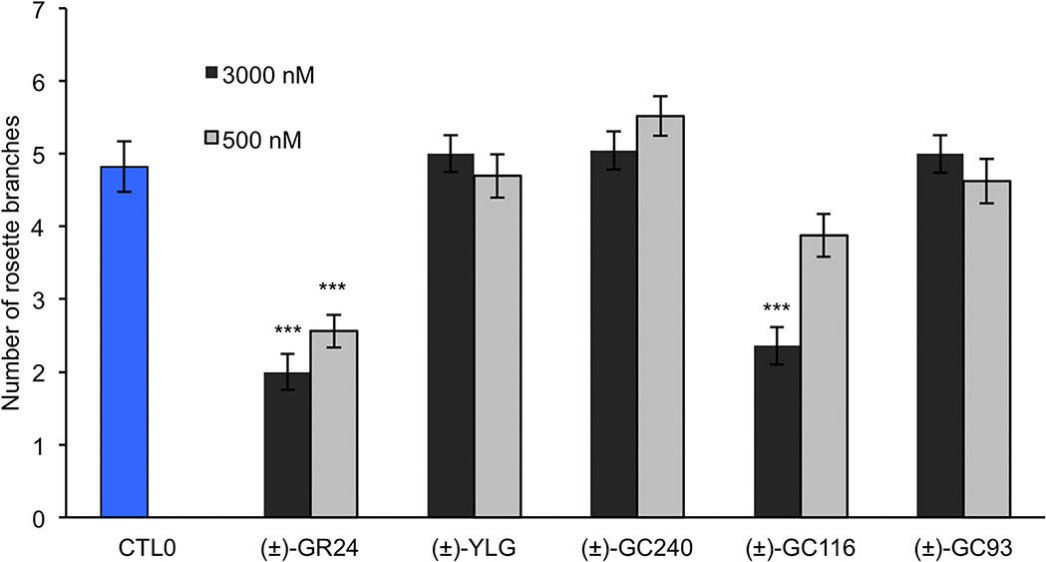
Bioactivity in Arabidopsis of probes vs. (±)-GR24 (*max3-11*). Number of rosette branches of mutant plants *max3-11* grown in long-day conditions. These data were obtained from means ± SE (*n* = 12 plants). ***P < 0.001 indicate significant differences with the control treatment (0 nM) (CTL0) (Kruskal–Wallis rank sum test). CTL0, control 0.

To compare the bioactivity of our probes between species, we performed a hydroponic bioassay with the Arabidopsis SL-deficient mutant *max3-11*. Since (±)-GC242 was previously found bioactive (de Saint Germain et al., 2016), we only tested the probes with one methyl group on the D-ring [(±)-GC240, (±)-GC116, (±)-GC93, and (±)-YLG] at two concentrations (0.5, 3 μM). The (±)-GR24 control treatment was bioactive at both concentrations whereas only the (±)-GC116 probe was found bioactive at 3 μM (Figure 6). This probe was also the most bioactive probe on pea for the control of shoot branching. In our conditions (±)-YLG and (±)-GC93 compounds were not bioactive contrary to previous studies (Tsuchiya et al., 2015; Wang et al., 2021). This result highlights the efficiency of our GC coumarin series on Arabidopsis and its appropriateness for *in vivo* investigations.

#### Coumarin and Resorufin Profluorescent Probes Are Bioactive in *P. patens*

**Figure 7:**
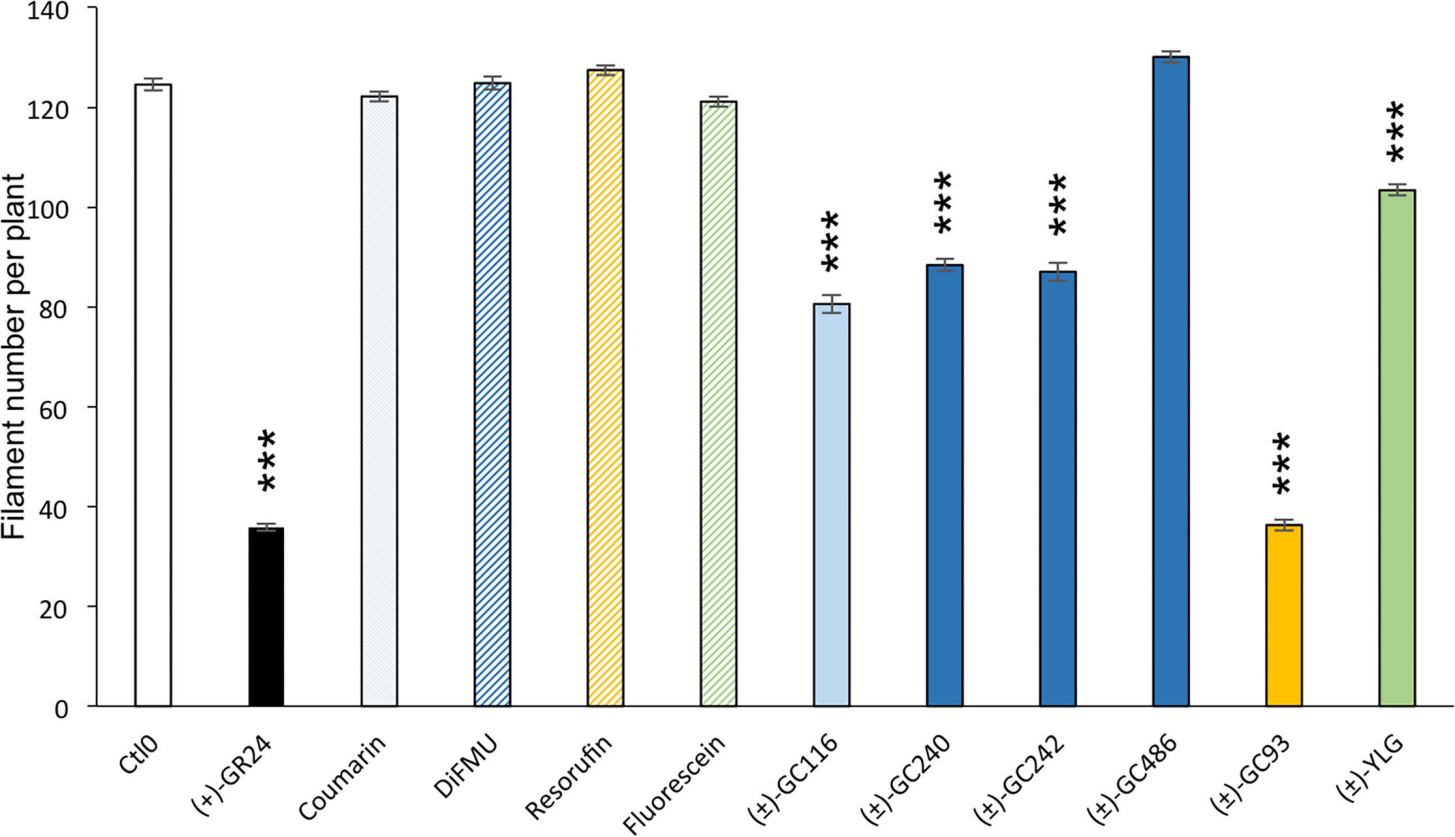
Probe bioactivity tested on the moss *P. patens*. Bioactivity of fluorophores and profluorescent probes was assayed by counting the number of filaments in the dark following application and was compared to that of DMSO (control 0, white), and (+)-GR24 (in black). Each compound was used at 1 μM. Fluorophore bars appear hatched, while the profluorescent probe bars appear plain. Data are mean ± SE of 24 *Ppccd8* mutant plants (*n* = 24) grown in three different 24-well plates. Significant differences between the control treatment (0 nM) and treated plants are based on ANOVA and Tukey's test as a *post-hoc* test; ***P < 0.001. CTL0, control 0.

In the moss *P. patens*, the biological activity of SL analogs was previously assayed by counting the number of filaments per plant, grown for 2 weeks in the dark following compound application (Guillory and Bonhomme, 2021). Both (±)-GR24 and (+)-GR24 enantiomer led to a decrease in filament number, in WT plants and in the *Ppccd8* mutant, where the activity was more pronounced (Hoffmann et al., 2014; Lopez-Obando et al., 2021). Using the two methyl profluorescent probe (±)-GC242 (Figure 2), a dose-dependent decrease in the filament number was observed in the *Ppccd8* mutant. However, the (±)-GC242 was found less active than (±)-GR24 (Lopez-Obando et al., 2021). We tested the activity of the GC series with only one methyl group and various fluorophores and compared it to that of (±)-GR24 and (±)-GC242. We also tested a profluorescent probe without a methyl group [(±)-GC486] since desmethyl GR24 was described as a better ligand for KAI2 in *Marchantia polymorpha*, which is another bryophyte (Yao et al., 2021; Figure 7). In the *Ppccd8* mutant, we first observed that none of the fluorophores had an effect on the filament number, and we confirmed the previous activity reported for (±)-GR24 and (±)-GC242. (±)-GC240 (one methyl group) had similar activity as (±)-GC242 (two methyl groups), while (±)-GC486 had a slight opposite effect on the number of filaments in one bioassay replication (Supplementary Figure 3). Thus, the presence/absence of a methyl group on the D-ring has a strong influence, but not the number of groups. All profluorescent probes with one methyl group but various fluorophores had a significant effect on the filament number. However, the strongest activity was observed with resorufin derivative (±)-GC93, while both coumarin probes [(±)-GC240 and (±)-GC116] showed similar moderate activities, and fluorescein probe (±)-YLG was found to be less active. In one bioassay replication, no significant bioactivity was detected for (±)-YLG and (±)-GC116 (Supplementary Figure 3). These data suggest that, in addition to the presence of methyl on the D-ring, the nature of fluorophore has an effect on the profluorescent probe activity in *P. patens*.

### Enzymatic Assays With the Profluorescent Probes

As previously described, after cleavage by the D14 proteins, these probes emit light when excited by a specific wavelength (Figure 3, Supplementary Figure 1). They allow us to have a quantitative follow-up of the reaction.

Two-phase cleavage kinetics was obtained with both (±)-GC242 and (±)-GC240: (1) an initial phase or burst phase corresponding to the fluorophore release during the first turnover (pre-steady state) and after a delay (few minutes or hours, depending on the ligand and the receptor); (2) a slow phase or a plateau (depending on the number of methyl on the D-ring) which can lead to return to the initial situation of a free D14 protein without ligand (steady state) for the probes bearing one methyl group. With two methyl group probes, a plateau was observed which does not allow for a second cleavage run for the protein, making this receptor unable to interact with other SLs (single turnover; de Saint Germain et al., 2016). We proposed that the different probes newly described, could be used to determine the parameters influencing the kinetic process and better understand the perception mechanism.

#### The Hydrolysis Kinetics by RMS3/PsD14 Are Different According to the GC Series Depending on the Number of Methyl Groups on the D-Ring

**Figure 8:**
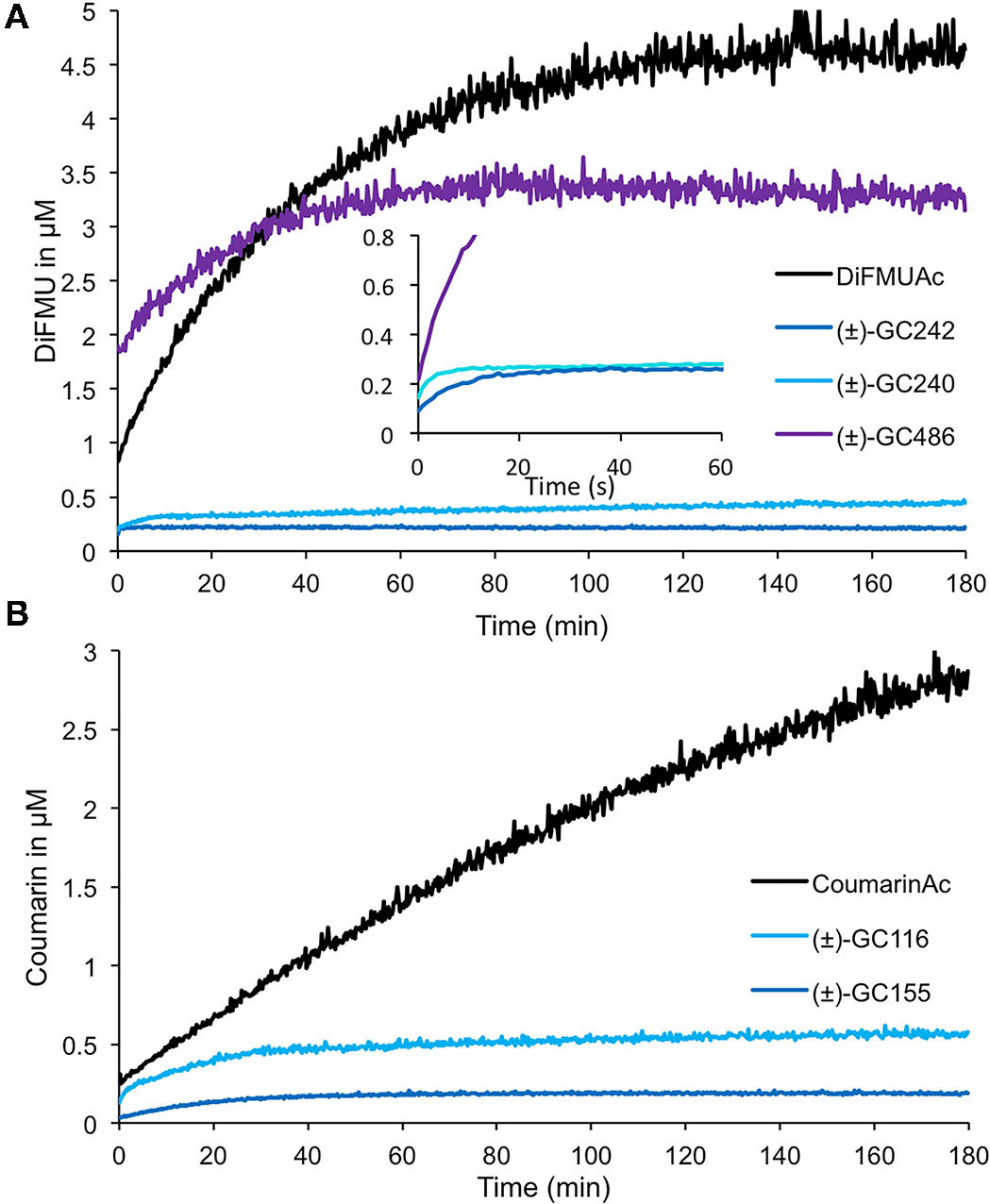
Enzymatic assays with profluorescent probes harboring various D-ring moieties or acetate groups. Progress curves during DiFMU **(A)** and Coumarin **(B)** profluorescent probes at 10 μM cleavage with RMS3 protein at 400 nM monitored (λ_em_: 460 nm) at 25°C. These traces represent one of the three replicates, and the experiments were repeated at least two times.

We performed enzymatic assays to study the effect of the D-ring structure on the kinetic cleavage. We used DiFMU probes harboring one [(±)-GC240], two [(±)-GC242], or no methyl group [(±)-GC486] on the D-ring, along with a molecule where the D-ring was replaced by an acetate group (DiFMU acetate, DiFMUAc; Figure 8A). We observed that the acetate probe kinetic differed from the other ones, with a higher extent of reaction but a slower reaction rate than those with one or two methyl groups. Moreover, the reaction seemed to be blocked at very low concentration for (±)-GC242 and (±)-GC240, in accordance with previous results (de Saint Germain et al., 2016). The (±)-GC486 kinetic differed from that of the other probes with a D-ring, with a high reaction rate, but the low slope of the cleavage kinetic curves during the initial phase in comparison to (±)-GC242 and (±)-GC240, suggests an initial slower cleavage velocity. We observed the same pattern with Coumarin acetate (CoumarinAc) vs. (±)-GC116 and (±)-GC155 (Figure 8B). However, CoumarinAc showed a slower velocity than DiFMUAc. The RMS3 showed Michaelian kinetics toward the acetate probes and (±)-GC486. Indeed, the hydrolysis of these probes was not blocked at a very low level, unlike for (±)-GC240 or (±)-GC242 (respectively due to the lack of D-ring or the absence of methyl group on the D-ring). This could be linked to the lack of bioactivity of these molecules on pea branching. Despite a higher velocity of DiFMUAc cleavage by RMS3, this probe shows the drawback to be poorly stable in PBS even in DMSO, in comparison to CoumarinAc (Supplementary Figure 4).

**Figure 9:**
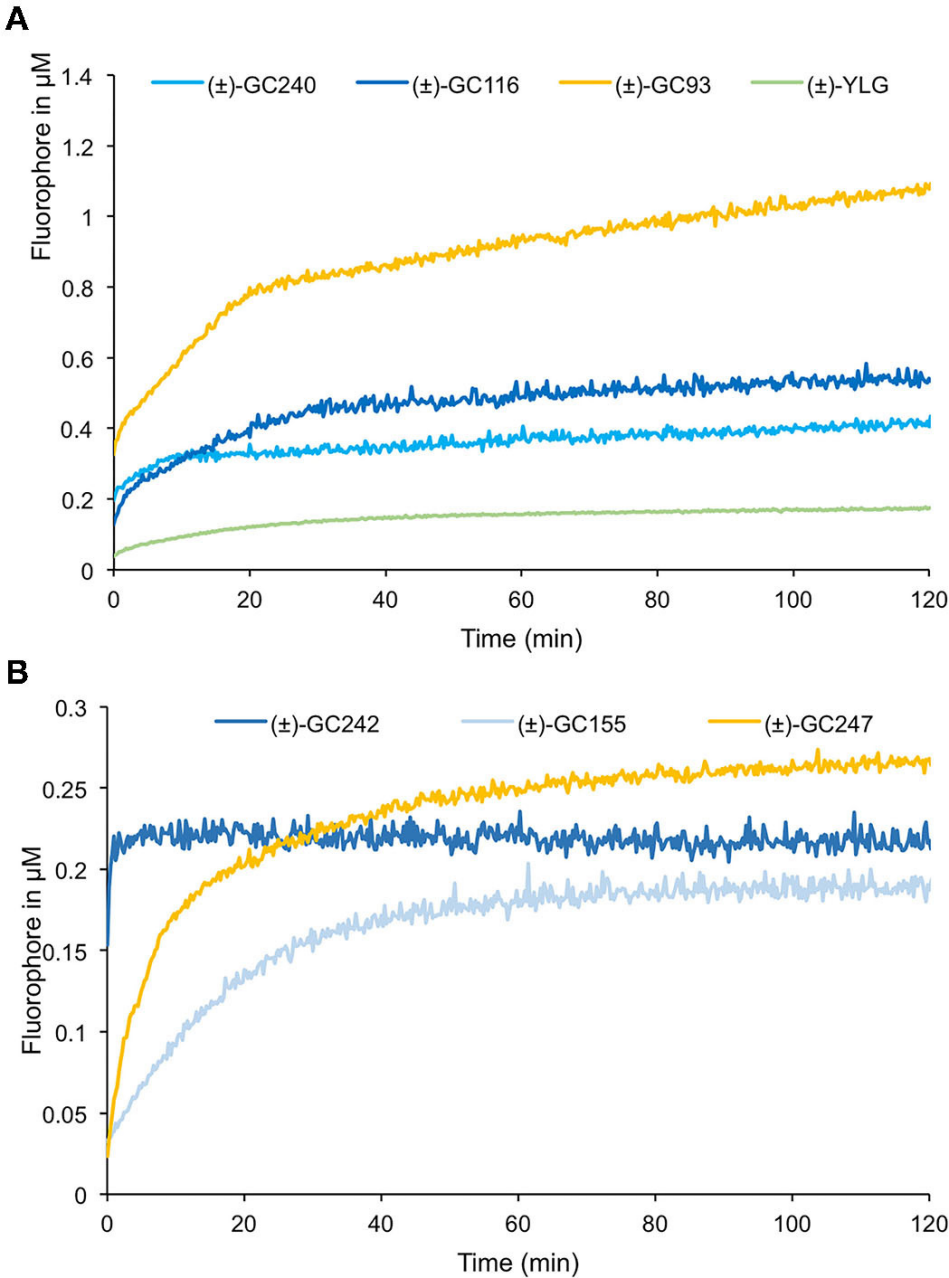
Enzymatic assays with profluorescent probes harboring various fluorophores. Progress curves profluorescent probes at 10 μM cleavage harboring D-ring with one methyl **(A)** or two methyl groups **(B)**, with RMS3 protein at 400 nM monitored (λ_em_: 460 nm) at 25°C. These traces represent one of the three replicates, and the experiments were repeated at least two times.

To study the effect of the cargo group on the SL cleavage kinetics, we compared (±)-GC242, (±)-GC155, and (±)-GC247 probes, harboring two methyl groups on the D-ring but having three different fluorophores (Figure 9) and for which a single turnover mechanism was proposed (de Saint Germain et al., 2016). By recording the fluorescence, we observed a two-phase kinetic for all three probes (Figure 9B), with a burst phase, or a presteady phase, followed by a steady phase where the product concentration reached a plateau as previously described with (±)-GC242. Looking at the slope of the presteady state for all the four probes [(±)-GC240, (±)-GC116, (±)-GC93 and (±)-YLG], we estimated that the enzymatic activity depended on the probe, and thus on the fluorophore molecule replacing the ABC-tricycle (Figure 9A). We speculated that the fluorophore group may mimic a cargo group that interacts with the binding pocket of RMS3 and may therefore, influence the affinity. On the contrary, the heights of the plateau values were all in the same range and did not seem to depend on the probe. These results support the hypothesis of a single turnover enzymatic mechanism for the probes with two methyl groups.

We performed similar assays for the probes with one methyl group on the D-ring and noticed a different kinetic mechanism (Figure 9A). We observed two steps: a very fast burst phase (<30 s), difficult to highlight in our conditions, followed by a slow phase (steady state) with no plateau, contrary to two methyl D-ring probes (Figure 9B). Indeed, in this second phase, the reaction did not seem to be blocked, but the velocity was very low, meaning that the reaction did not perfectly follow a single turnover mechanism. Presumably, some RMS3 protein might catalyze more than one probe molecule. We also observed differences between the progress curves of the different probes, meaning that the cargo group still had an influence on the enzymatic mechanism.

To search for a destabilization effect, which characterizes bioactive SL analogs with SL receptors, we performed DSF binding assay with our novel probes on RMS3 protein. We confirmed that the (±)-GC116, (±)-GC155, and (±)-GC379 probes were able to destabilize RMS3 (Supplementary Figure 5). Similar investigations were not possible with resorufin and fluorescein probes [(±)-GC93, (±)-GC247, (±)-YLG] due to the overlap of their emission spectra with that of SYPRO^TM^ orange and RMS3 (Figure 3). We noticed three different behaviors for the probes with a D-ring according to their number of methyl groups. The (±)-GC486, with no methyl group on the D-ring, did not show a single turnover kinetic, but more likely a curve that resembled that of the acetate probes. The probes with two methyl groups showed a rapid and blocked enzymatic reaction that fits with the hypothesis of a single turnover mechanism. Finally, the probes with one methyl group had a particular kinetic that could be partly linked to a single turnover mechanism. These assays suggest that the number of methyl groups is important for covalent adduct stability.

#### Comparison of the Hydrolysis Kinetics Between SL Receptor From Different Species

**Figure 10:**
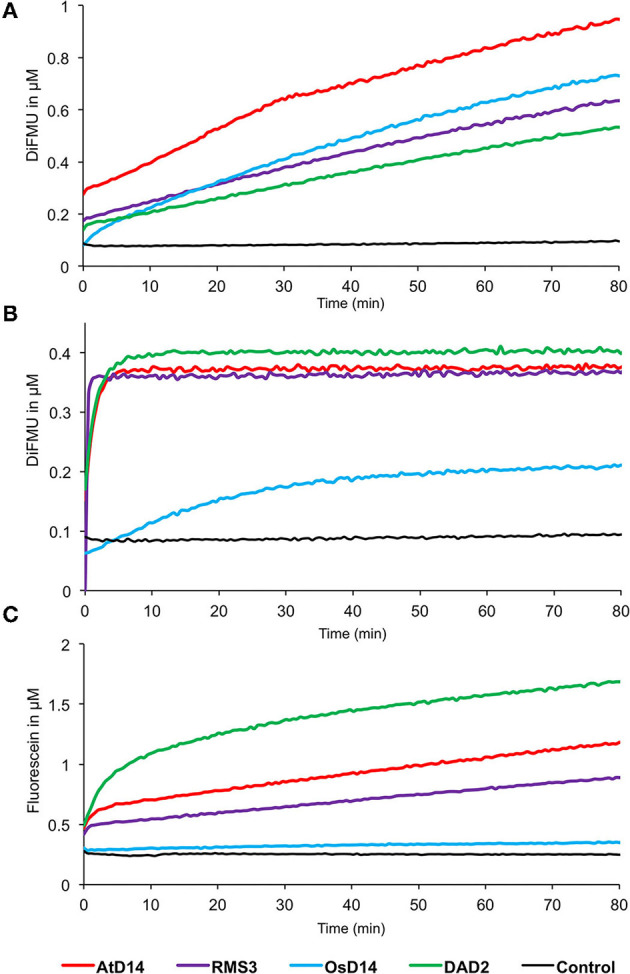
Enzymatic kinetics for AtD14, RMS3, OsD14, and DAD2 proteins incubated with (±)-GC240 **(A)**, (±)-GC242 **(B)**, or (±)-YLG **(C)**. Progress curves during probes cleavage, monitored (λ_em_ 460 nm for (±)-GC240 and (±)-GC242 and λ_em_ 510 nm for (±)-YLG) at 25°C. Protein catalyzed cleavage with 400 nM of protein and 20 μM of probes. These traces represent one of the three replicates, and the experiments were repeated at least two times.

Finally, the GC probes were used to compare the enzymatic activity of RMS3, AtD14, DAD2, and OsD14 proteins. We compared the enzymatic kinetics of these proteins at a concentration of 0.33 μM toward three different probes at 10 μM [(±)-GC240, (±)-GC242, and (±)-YLG, Figure 10]. All tested proteins were able to cleave the (±)-GC240 and (±)-GC242 probes but differences in the reaction kinetics were observed. With (±)-GC240 cleavage, it was highly difficult to highlight the rapid phase of the kinetic (due to the low time resolution), except for OsD14 suggesting a lower affinity of the rice SL receptor toward (±)-GC240 (Figure 10A), confirmed by the cleavage profile of (±)-GC242 (Figure 10B). Surprisingly, we observed that OsD14 was unable to cleave (±)-YLG, in contrast to the three other proteins (Figure 10C).

## Discussion

### Importance of Having Probes With Different Spectral Properties

The design of profluorescent SL probes was focused on obtaining bioactive molecules with spectral properties compatible with biochemistry approaches, such as enzymatic kinetics and fluorescence-based binding assays (DSF, nanoDSF, or intrinsic fluorescence assays). Probes should present a high molecular brightness and a large Stokes shift to easily record the fluorescence emission with classical equipment. Probes also need to be highly stable to perform kinetic measurements. Unfortunately, none of the molecules tested here could combine all these properties, for example, resorufin. Resorufin is a common fluorophore used in profluorescent probes (Gao et al., 2003; Zhang et al., 2015; Yan et al., 2016; Biswas et al., 2017; Wu et al., 2017; Tian et al., 2021). It showed a high brightness, a broad spectrum, and longer analytical wavelength than the fluorescein moiety present in YLG, efficient for *in planta* imaging and has been claimed (Wang et al., 2021) to outperform YLG series, for its optical properties more adapted to *in planta* imaging. However, resorufin probes present small Stokes shifts, which are the major limitations of resorufin series, and are not suitable for DSF assays. An opportunity in the development of efficient SL profluorescent probes focussed not only on the modulations of resorufin unit to improve the p*K*_*a*_, solubility and the membrane permeability but also on expanding the Stokes shifts as recently reported (Tan et al., 2021). Thus, the development of novel profluorescent SL should offer tools for dedicated applications.

### Important Effect of Chemical Structures of Profluorescent Probes for Bioactivity

In comparison to (±)-YLG, the GC probes showed lower brightness, which is a drawback of fluorescent detection, but with the leaving group, the GC probes showed a hindrance more similar to that of natural SLs. Accordingly, we found out that GC probes were biologically active in pea, with a better bioactivity for coumarin-based probes. Only the most active probes in pea [(±)-GC242 and (±)-GC116] were significantly bioactive in Arabidopsis. We demonstrated that the coumarin profluorescent probes were highly bioactive and well-adapted to dissect the enzymatic properties of SL receptors. The high bioactivity of GC coumarin probes is linked not only to their hydrophobicity (Log*P*) close to that of (±)-GR24 but also to the good cleavability of the leaving groups in relation to their low p*K*_*a*_ values (Figure 2) as noticed for debranones (Fukui et al., 2017). This high bioactivity could also be attributed to their binding affinity to SL receptors, mainly based on the cargo group (i.e., fluorescent part). Our experimental data are consistent with a recent molecular simulation study (Wang et al., 2021). Based on the demonstrated high bioactivity of different SL coumarin profluorescent probes in vascular plants, we can assert that this chemical backbone constitutes a relevant working basis for developing new probes with refined properties. Coumarin is a fluorophore that has been repeatedly used to design sensors aiming at the detection of biological elements and phenomena of many different origins (Cao et al., 2019). It is reported in several studies that can easily inspire us in this quest. Further design on the fluorophore backbone itself, in order to adjust its optical properties (brightness, absorption, and emission wavelengths) and/or physicochemical properties (solubility, p*K*_*a*_, log*P*) may also allow us to develop new molecules that are more relevant for use in biological environments (Roubinet et al., 2015).

### Profluorescent Probes: Clues for Knowledge in SL Perception in Pea and Arabidopsis

Enzymatic competition assay with YLG and GC probes have been used to characterize the perception mechanism of newly identified D14 ligand. However, the interpretation of these results and the determination of kinetic constant like *K*_i_ (inhibition constant) remains challenging because D14 does not behave like a Michaelian enzyme toward these probes. To overcome this difficulty, it is possible to use the acetate probes to perform enzymatic competition assay and characterize more easily the type of competition mechanism and compare different ligand binding properties.

We observed that some of these probes are not only hydrolyzed by D14 proteins but are also not biologically active on pea [i.e., DiFMUAc, coumarinAc, and (±)-GC486]. This means that the bioactivity does not depend on the cleavage of the molecules, but more probably on the formation of a particular intermediate. The biological activity is also dependent on the presence of D-ring with one or two methyl groups, which suggests that this part of the molecule participates in the perception mechanism. Different parameters influence the affinity and kinetics of plant SL receptors in the presence of SLs: they depend both on the D-ring and on the cargo group. The cargo group, which corresponds to ABC-tricycle in canonical SLs, is partially responsible for the interaction with D14. Thus, it could influence the reaction rate and the apparent affinity because this part of the molecule acts in the first contact with D14. Moreover, the structure of the D-ring part also influences the enzymatic mechanism as it was observed with the variation of the number of methyl groups. Indeed, the probes with two methyl groups seemed to undergo a strict single turnover mechanism while those with one methyl group showed a burst phase followed by a slow phase. The covalent adduct created with the D-ring with two methyl groups could be more stable due to steric interactions and/or electronic effects in contrast to the D-ring with one methyl group and even with no methyl group for which no covalent adduct was detected with RMS3 (de Saint Germain et al., 2016). To precisely compare the enzymatic activity of the different receptors toward each probe, and to provide a better understanding of SL perception mechanism, it is necessary to determine kinetic constants like *K*_M_, *V*_max_, and *k*_cat_. Since it is clear that this mechanism depends on the structure of the SL molecule, it could be interesting to modulate p*K*_*a*_, hindrance, and hydrophobicity of the probes to link cleavage kinetics, bioactivity, and perception mechanism.

### Profluorescent Probes: Tools to Perform SAR Study and Compare Bioactivity Between Species

We have shown that the hydrolysis profile of profluorescent probes is not only dependent on the probes but also on the SL receptors. There is generally a correlation between a fast cleavage of the probe and a good biological activity on pea and Arabidopsis. This should be verified not only for petunia but also for rice for which OsD14 protein is not able to cleave (±)-YLG.

Furthermore, the hydrolysis activity is proposed to be determinant to have a highly sensitive SL receptor as in *Striga, Orobanche*, and *Phelipanche* (de Saint Germain et al., 2021b; Chen et al., 2022). GC and (±)-YLG probes showed germination activity in these parasitic plant seeds but much weaker than SLs and without selectivity (de Saint Germain et al., 2021b; Wang et al., 2021). SL profluorescent probes with better efficiency would be worth being developed for the study of SL receptors in these plants.

In *P. patens* where there is no D14 ortholog, 13 *PpKAI2Like* genes have been reported as encoding candidate receptors for SL and for the so far unknown KAI2-Ligand (KL). Strikingly, the SL and the KL pathways have opposite effects on the filament number and the phenotype assayed in the present study. The (+)-GR24 is a good mimic for SL in moss, decreasing the number of filaments, and is likely to be perceived by the PpKAI2L (GJM) clade (Lopez-Obando et al., 2021). Here, we show that (±)-GC93 has the best bioactivity as SL mimics in *P. patens*, being even more potent than (±)-GC242. This profluorescent probe could thus be used to further analyze SL perception mechanism in moss, when PpKAI2L-G,J recombinant proteins will be available (Lopez-Obando et al., 2021). Besides, the (−)-GR24 has proven as a poor mimic for studying the KL pathway by the PpKAI2L (A-E) clade. Although the natural SLs have only one methyl group on the D-ring (Yoneyama, 2020), recent results demonstrated that (−)-desmethyl GR24 was a better mimic of KAI2-ligands (KL) than (−)-GR24 (Yao et al., 2021). In one assay reported above, the (±)-GC486 (no methyl on the D-ring) showed an opposite effect to that of other probes, increasing the number of filaments (Supplementary Figure 3). The (±)-GC486 thus needs to be tested as a potential KL agonist on moss WT and *Ppkai2La-e* mutants (Lopez-Obando et al., 2021).

### Tools for new Investigation/Applications

#### Research of Agonists and Antagonists With Profluorescent Probes

Synthetic inhibitors KK094 (Nakamura et al., 2019), TFA (Hamiaux et al., 2018), and DL1b (Yoshimura et al., 2020) of D14 SL receptors have been described in Arabidopsis and petunia. Their discovery was based especially on their aptitude to inhibit the hydrolysis of (±)-YLG in competition assays with SL receptors. However, no bioactivity of these molecules (KK094, TFA, DL1b) was detected in pea. A screen of chemical libraries for potential SL agonists and antagonists could thus be undertaken using our GC coumarin tools [e.g., (±)-GC242 or (±)-GC116] highly bioactive in pea for bud outgrowth inhibition *via* RMS3, to discover novel hits. With the GC probes, it would be also possible to characterize OsD14 enzymatic properties and screen for compounds interacting with the SL rice receptor, that is not possible with (±)-YLG. The use of different fluorophores could facilitate high throughput screening for active molecules and inhibitor, especially to detect molecules with fluorescence property that perturb the signal detection and are therefore used to be eliminated from the screen.

The (±)-YLG has also been used to validate SL receptor agonists (Uraguchi et al., 2018) or antagonists (Holbrook-Smith et al., 2016; Arellano-Saab et al., 2022; Zarban et al., 2022) for *Striga*. Again, the discovery of a profluorescent probe, which is as active as SLs, remains to be discovered to obtain a more relevant screening tool for the discovery of efficient inhibitors for SL receptors or SL mimics.

#### Characterization of Other Enzymes

Very recently, a degradation pathway for SLs has been discovered in *Arabidopsis thaliana* (Xu et al., 2021). It involves a carboxylesterase (AtCXE15), with no SL reception function, which was demonstrated to be able to break SL molecules and thereby modulate shoot branching. The SL profluorescent probes are also very promising tools to characterize this type of enzyme or any protein that is able to cleave SLs.

#### For in Planta Imaging

Fluorogenic SL probes are essential tools for *in planta* imaging, but tissue autofluorescence is a major problem in plants, due to the high content of photosynthetic pigments. With the expansion of profluorescent probes repertory, it would be possible to develop microscopy imaging specifically to localize SL perception. Co-localization with GFP-tagged proteins would also be easier with GC probes, while fluorescein spectra overlapping with GFP prevents such studies (Figure 3).

## Conclusion

To conclude, our experiments partially unveiled the complexity and the diversity of SL perception by the D14 family of receptors. We emphasized that no profluorescent SL probe was universal and that these probes should be used with caution depending on their designated purpose. Our molecular tools described could help to discover novel useful agonists/antagonists of SL receptors for applications and fundamental knowledge.

## Data Availability Statement

The raw data supporting the conclusions of this article will be made available by the authors, without undue reservation.

## Author Contributions

F-DB, AdSG, SB, and CR designed the research. GC and SDF designed and synthesized the probes. F-DB performed the HPLC analyses. F-DB, J-PP, AG, A-VS, and SB performed the biological experiments. AdSG and PS performed the biochemical experiments. AC recorded the fluorescent spectra. AdSG, SB, CR, and F-DB wrote the paper. All authors analyzed the data. All authors critically revised the manuscript. All authors contributed to the article and approved the submitted version.

## Funding

We are grateful to the Agence Nationale de la Recherche (contracts ANR-12-BSV6-0004-01 and ANR-21-CE20-0026-04) for financial support. The IJPB benefits from the support of Saclay Plant Sciences-SPS (ANR-17-EUR-0007). This work has benefited from the support of IJPB's Plant Observatory technological platforms. AdSG has received the support of the EU in the framework of the Marie-Curie FP7 COFUND People Programme, through the award of an AgreenSkills/AgreenSkills+ fellowship and the support of Saclay Plant Sciences-SPS (ANR-17-EUR-0007) through the award of a fellowship. The CHARM3AT LabEx program (ANR-11-LABX-39) is also acknowledged for its support.

## Conflict of Interest

The authors declare that the research was conducted in the absence of any commercial or financial relationships that could be construed as a potential conflict of interest.

## Publisher's Note

All claims expressed in this article are solely those of the authors and do not necessarily represent those of their affiliated organizations, or those of the publisher, the editors and the reviewers. Any product that may be evaluated in this article, or claim that may be made by its manufacturer, is not guaranteed or endorsed by the publisher.
