# Supplementary material for: Expansion of the Strigolactone Profluorescent Probes Repertory: The Right Probe for the Right Application
Source: Front Plant Sci. 2022 Jun 2;13:887347. doi: 10.3389/fpls.2022.887347 (PMC9201908; doi:10.3389/fpls.2022.887347)
Supplement: Supplementary file 1 [file Data_Sheet_1.pdf]

Supplementary Material

Expansion of the strigolactone profluorescent probes repertory: the right probe for the right application

Alexandre de Saint Germain[1], Guillaume Clavé[2], Paul Schouveiler[1], Jean-Paul Pillot[1], Abhay-Veer Singh[1], Arnaud Chevalier[2], Suzanne Daignan Fornier[2], Ambre Guillory[1], Sandrine Bonhomme[1], Catherine Rameau[1], François-Didier Boyer[2*]

[1] Université Paris-Saclay, INRAE, AgroParisTech, Institut Jean-Pierre Bourgin (IJPB), 78000, Versailles, France

[2] Université Paris-Saclay, CNRS, Institut de Chimie des Substances Naturelles, UPR 2301, 91198, Gif-sur-Yvette, France

Supplementary Tables

Compound (no. of replicates) [a]	Concentration	Length of bud at node 3 /branch (mm) [b]	SE	p-value [d]
GC116	10,000 nM	2.43	0.12	0.000000e+00
	1,000 nM	1.67	0.09	0.000000e+00
	1,000 nM	3.13	1.43	0.000000E+00
	1,000 nM	1.60	0.08	0.000000e+00
	1,000 nM	2.22	0.19	0.000000e+00
	1,000 nM	1.97	0.09	0.000000e+00
	1,000 nM	2.21	0.07	0.000000e+00
	100 nM	1.96	0.11	0.000000e+00
	100 nM	1.72	0.11	0.000000e+00
	100 nM	2.66	0.26	2.084775e-10
	100 nM	2.29	0.09	0.000000e+00
	100 nM	2.46	0.15	0.000000e+00
	10 nM	2.47	0.19	3.762324e-12
	10 nM	2.26	0.20	0.000000e+00
	10 nM	5.20	1.48	3.729298e-04
	10 nM	4.34	0.66	0.000000e+00
	10 nM	3.85	0.63	0.000000e+00
	1 nM	6.38	1.09	1.221245e-15
	0.1 nM	11.47	1.40	1.464535e-01

GC155	10,000 nM	2.06	0.10	0.000000e+00
	1,000 nM	1.98	0.23	0.000000e+00
	1,000 nM	1.45	0.10	0.000000e+00
	1,000 nM	1.92	0.11	2.231548e-14
	1,000 nM	1.96	0.11	0.000000e+00
	1,000 nM	2.38	0.14	0.000000e+00
	1,000 nM	2.32	0.11	0.000000e+00
	100 nM	5.98	2.00	2.793725e-03
	100 nM	1.83	0.31	0.000000e+00
	100 nM	3.68	0.87	8.323844e-02
	100 nM	4.11	1.00	0.000000e+00
	100 nM	2.71	0.13	0.000000e+00
	10 nM	5.97	1.73	3.518254e-04
	10 nM	16.85	6.28	1.581675E-02
	10 nM	8.43	1.42	0.000000e+00
GC379	10,000 nM	2.17	0.09	0.000000e+00
	1,000 nM	3.38	0.73	2.260369e-01
	1,000 nM	3.19	0.23	0.000000e+00
	1,000 nM	3.65	0.87	0.000000e+00
	1,000 nM	3.04	0.81	0.000000e+00
	100 nM	2.81	0.54	5.485758e-04
	100 nM	5.02	1.14	0.000000e+00
	100 nM	3.98	0.93	0.000000e+00
	100 nM	14.32	3.98	7.197213e-01

	10 nM	3.54	0.93	0.000000e+00
GC380	10,000 nM	2.15	0.11	0.000000e+00
	1,000 nM	2.33	0.23	9.998758e-01
	1,000 nM	3.47	1.02	0.000000e+00
	1,000 nM	2.08	0.16	0.000000e+00
	100 nM	8.99	1.78	2.013884e-07
	100 nM	11.66	1.93	3.914880e-05
	100 nM	5.90	1.34	0.000000e+00
	10 nM	6.65	1.39	0.000000e+00
GC247	10,000 nM	6.54	1.01	5.605286e-03
	1,000 nM	26.93	2.14	7.244459e-01
	1,000 nM	18.57	1.13	6.735066e-01
	1,000 nM	16.56	1.36	0.000000e+00
	100 nM	15.23	1.46	1.000000e+00
	100 nM	14.78	2.88	2.188517e-01
	100 nM	11.82	1.21	6.683622e-02
	10 nM	12.39	1.20	5.732650e-02
GC93	10,000 nM	3.89	0.55	3.938098e-06
	1,000 nM	21.70	2.48	3.400014e-01
	1,000 nM	10.23	1.34	1.629355e-01
	1,000 nM	11.80	1.30	1.516977e-01
	100 nM	20.83	0.66	1.231200e-02
	100 nM	17.20	1.06	9.999864e-01

	10 nM	17.73	1.10	1.000000e+00
YLG	10,000 nM	2.44	0.43	0.000000e+00
	10,000 nM	4.81	1.02	0.000000e+00
	1,000 nM	7.40	1.87	7.241036e-01
	1,000 nM	15.90	1.79	5.498252e-01
	1,000 nM	3.40	0.68	3.165924e-09
	1,000 nM	5.59	1.10	0.000000e+00
	1,000 nM	14.28	1.97	1.990799e-08
	100 nM	5.27	1.13	4.097332e-02
	100 nM	19.15	1.45	1.000000e+00
	100 nM	8.39	1.68	1.887069e-02
	100 nM	22.73	1.33	9.953965e-01
	100 nM	19.89	2.35	2.426501e-01
	10 nM	24.15	0.98	7.806233e-01
GC240	5,000 nM[c]	2.61	0.23	0.000000e+00
	1,000 nM	14.17	2.02	9.999973e-01
	1,000 nM	6.22	2.01	3.78319e-09
	1,000 nM	5.64	0.85	0.000000e+00
	1,000 nM[c]	2.52	0.25	0.000000e+00
	1,000 nM	6.85	1.05	0.000000e+00
	100 nM	17.88	1.79	9.552628e-01
	100 nM	10.78	1.60	2.039812e-01
	100 nM	17.14	1.35	2.764565e-10
	10 nM	19.68	1.67	4.675064e-01

	10 nM	15.01	1.39	9.998463e-01
	10 nM	22.08	1.91	5.122958e-01
GC486	5,000 nM	14.02	1.80	3.208221e-01
	5,000 nM	7.80	1.61	5.733226E-01
	5,000 nM[c]	17.41	2.10	9.763187e-01
	1,000 nM[c]	9.26	2.37	2.647994e-01
	1,000 nM	23.14	0.94	1.912903e-01
	100 nM	22.10	1.39	7.608111e-02
	10 nM	24.70	1.12	9.932760e-01
GC242	10,000 nM	1.77	0.07	0.000000e+00
	1,000 nM	2.09	0.11	0.000000e+00
	1,000 nM	3.26	0.61	3.554379e-12
	1,000 nM	1.78	0.10	0.000000e+00
	1,000 nM	2.25	0.28	0.000000e+00
	1,000 nM	1.64	0.07	0.000000e+00
	1,000 nM	1.40	0.04	0.000000e+00
	1,000 nM	1.70	0.08	0.000000e+00
	1,000 nM	1.98	0.08	0.000000e+00
	100 nM	2.02	0.08	0.000000e+00
	100 nM	1.80	0.08	0.000000e+00
	100 nM	4.33	1.37	3.508555e-08
	100 nM	4.20	1.73	0.000000e+00
	100 nM	1.75	0.13	0.000000e+00

	100 nM	1.66	0.06	0.000000e+00
	100 nM	2.05	0.10	0.000000e+00
	10 nM	9.36	1.88	2.622541e-01
	10 nM	6.13	1.18	0.000000e+00
7-Acetoxycoumarin	1,000 nM	25.33	1.02	9.998766e-01
	100 nM	22.67	1.91	8.566230e-01
	10 nM	21.81	1.30	6.403749e-02
DIFMUA	1,000 nM	22.17	1.24	1.051261e-01
	100 nM	19.75	1.61	1.669144e-03
	10 nM	23.71	1.70	9.895969e-01
7-hydroxycoumarine	1,000 nM	41.46	9.43	9.991303e-01
DiFMU	10,000 nM	19.07	0.99	7.238675e-01
	10,000 nM	7.67	1.89	6.997665e-01
	5,000 nM	16.25	0.65	9.999711e-01
	5,000 nM	8.44	1.58	6.997665e-01
	1,000 nM	14.76	1.77	7.908909e-01
Resorufin	1,000 nM	14.90	1.13	9.899278e-01
	100 nM	16.50	0.80	9.999784e-01
	10 nM	12.53	1.15	1.447628e-01

Supplementary Table 1. Bud outgrowth inhibition activity assay results for SL probes and derivatives by direct application on *rms1-10* pea mutant plants. [a] All the replicates are presented in Supplemental Table 1. [b] These data were obtained from means ± SE (n = 24), 10 days after treatment. [c] Tested previously (De saint Germain et al 2016). [d] Comparison of the SL treatment to the control treatment (0 nM) using the Kruskal-Wallis rank sum test.

Compound (no. of replicates) [a]	Concentration	Length of bud at node 3 /branch [b]	SE	p-value [c]
GC379	1,000 nM	26.30	0.70	5.353000e-01
GC380	1,000 nM	23.62	1.63	5.353000e-01
GC242	5,000 nM	33.76	2.19	7.156579e-01
	1,000 nM	35.80	1.49	9.991219e-01

Supplementary Table 2. Bud outgrowth inhibition activity assay results for SL probes by direct application on *rms3-4* pea mutant plants. [a] All the replicates are presented in Supplemental Table 2. [b] These data were obtained from means ± SE (n = 24), 10 days after treatment. [c] Comparison of the SL treatment to the control treatment (0 nM) using the Kruskal-Wallis rank sum test.

Compound (no. of replicates) [a]	Concentration	Length of bud at node 3, 4, 5 /branch (mm) [b]	SE	p-value [f]	p-value [g]	p-value [h]	p-value [i]
GC242	3,000 nM	1.90[c]	0.12	0.000000e+00			
	3,000 nM	2.11[c]	0.14	0.000000e+00		9.999997e-01	0.000000e+00
	3,000 nM	1.26[d]	0.04	0.000000e+00			
	3,000 nM	1.15[d]	0.10	0.000000e+00		6.623814e-03	
	3,000 nM	0.98[d]	0.03	0.000000e+00		6.847110e-06	1.712978e-02
	3,000 nM	1.01[e]	0.05	0.000000e+00			
	3,000 nM	0.71[e]	0.11	0.000000e+00		0.000000e+00	
	3,000 nM	0.76[e]	0.04	0.000000e+00		0.000000e+00	0.000000e+00
YLG	3,000 nM	6.24[c]	2.06	4.097445e-04	3.046445e-02		
	3,000 nM	4.04[c]	0.45	1.469103e-11	6.026631e-01	6.641796e-01	0.000000e+00
	3,000 nM	3.17[d]	1.06	7.253980e-05	4.463705e-03		
	3,000 nM	3.36[d]	1.24	4.647728e-01	1.420836e-04	9.805131e-01	
	3,000 nM	2.07[d]	0.59	1.262800e-03	8.146868e-02	9.902806e-01	9.953035e-01
	3,000 nM	3.25[e]	0.57	5.912796e-05	0.000000e+00		
	3,000 nM	1.91[e]	0.40	4.303795E-01	0.000000e+00	9.996235e-01	
	3,000 nM	1.46[e]	0.14	0.000000e+00	0.000000e+00	9.999605e-01	1.849925e-01
GC93	3,000 nM	11.61[c]	3.82	4.954004e-01		4.909945e-01	
	3,000 nM	3.96[d]	1.14	1.170460e-04		4.122257e-01	

Supplementary Table 3. Bud outgrowth inhibition activity assay results for SL probes and derivatives by vascular supply on *rms1-10* pea mutant plants. [a] All the replicates are presented in Supplemental Table 3. [b] These data were obtained from means ± SE (n = 12), 10

days after treatment. [c] Node 3. [d] Node 4. [e] Node 5. [f] Comparison of the SL treatment to the control treatment (0 nM) using the Kruskal-Wallis rank sum test. [g] Comparison of the SL treatment to (±)-GC242 treatment (3000 nM) using the Kruskal-Wallis rank sum test. [h] Comparison of the SL treatment to (±)-GR24 treatment (3000 nM) using the Kruskal-Wallis rank sum test. [i] Comparison of the SL treatment to wild type Térèse non treated using the Kruskal-Wallis rank sum test.

frontiers

A

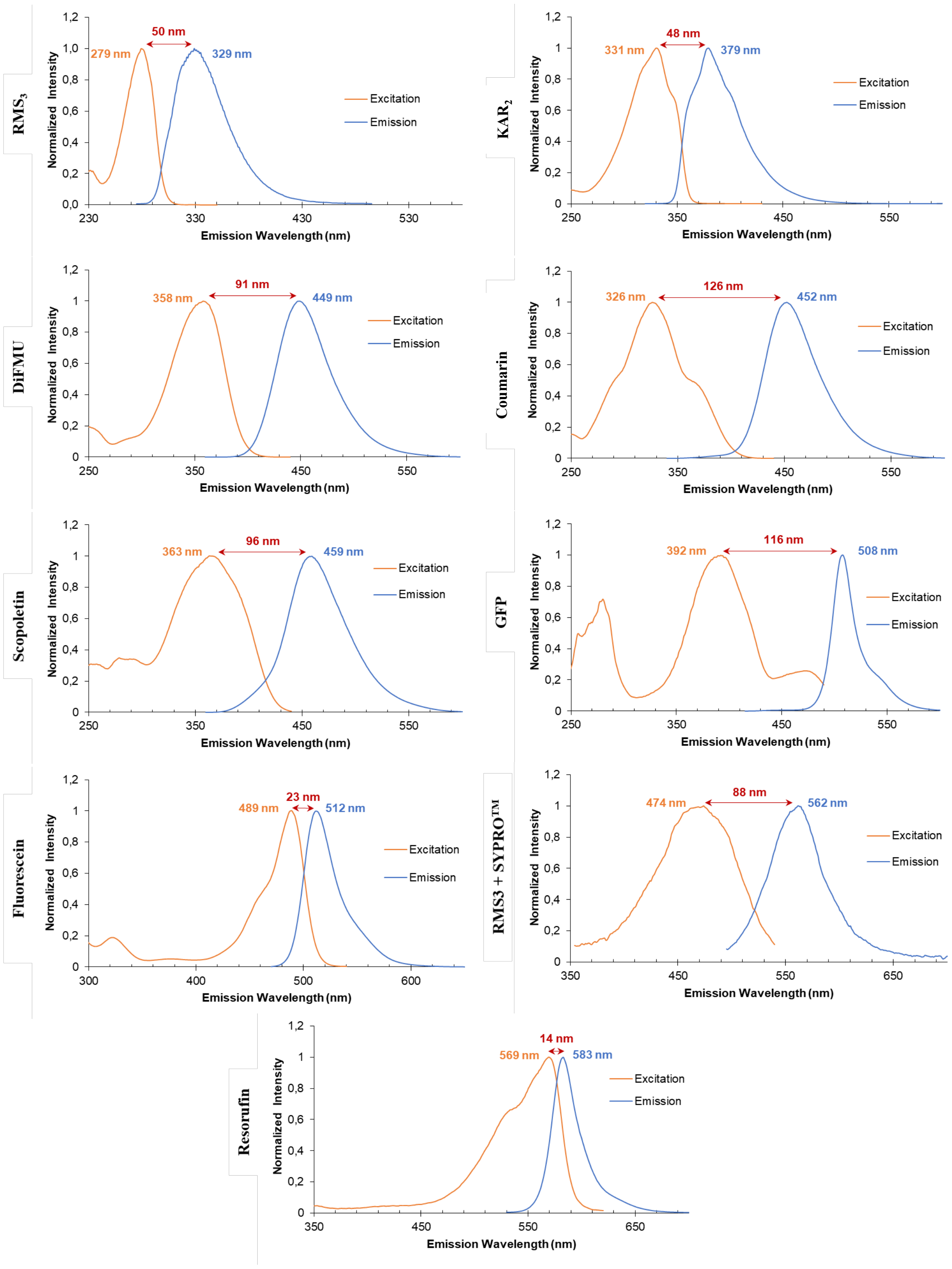

RMS3
Normalized Intensity
Emission Wavelength (nm)
279 nm
50 nm
329 nm
Excitation
Emission
KAR2
331 nm
48 nm
379 nm
DiFMU
358 nm
91 nm
449 nm
Coumarin
326 nm
126 nm
452 nm
Scopoletin
363 nm
96 nm
459 nm
GFP
392 nm
116 nm
508 nm
Fluorescein
489 nm
23 nm
512 nm
RMS3 + SYPRO™
474 nm
88 nm
562 nm
Resorufin
569 nm
14 nm
583 nm

B

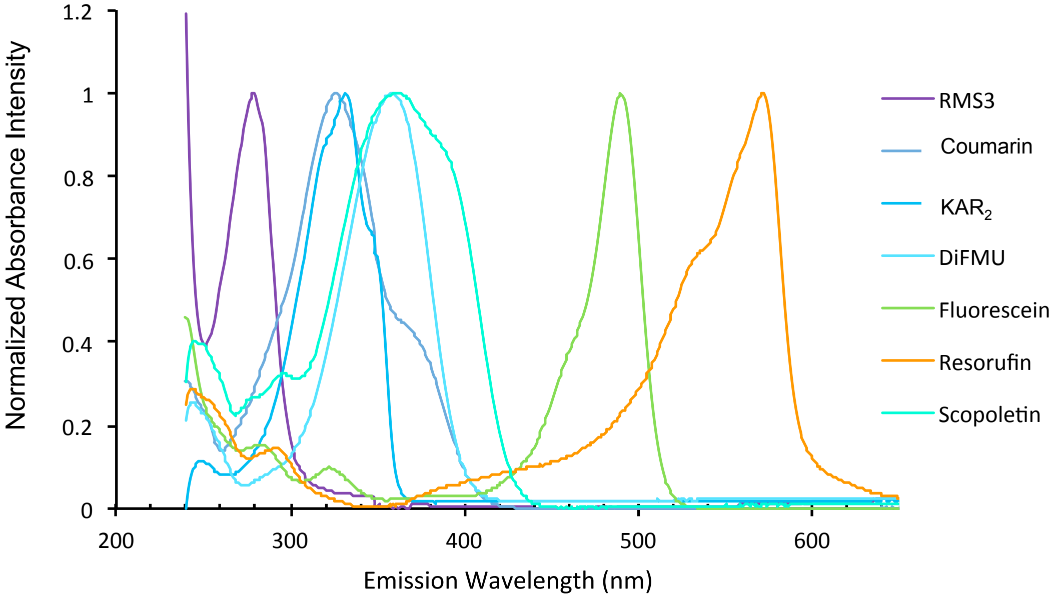

Supplemental Figure 1. Fluorescence emission and excitation spectra recorded in PBS Buffer pH = 7.6 of various fluorophores and RMS3 protein at 25 °C (**A**). Superposition of absorbance spectra recorded in PBS Buffer pH = 7.6 at 25 °C (**B**). The Stokes shift is indicated in red.

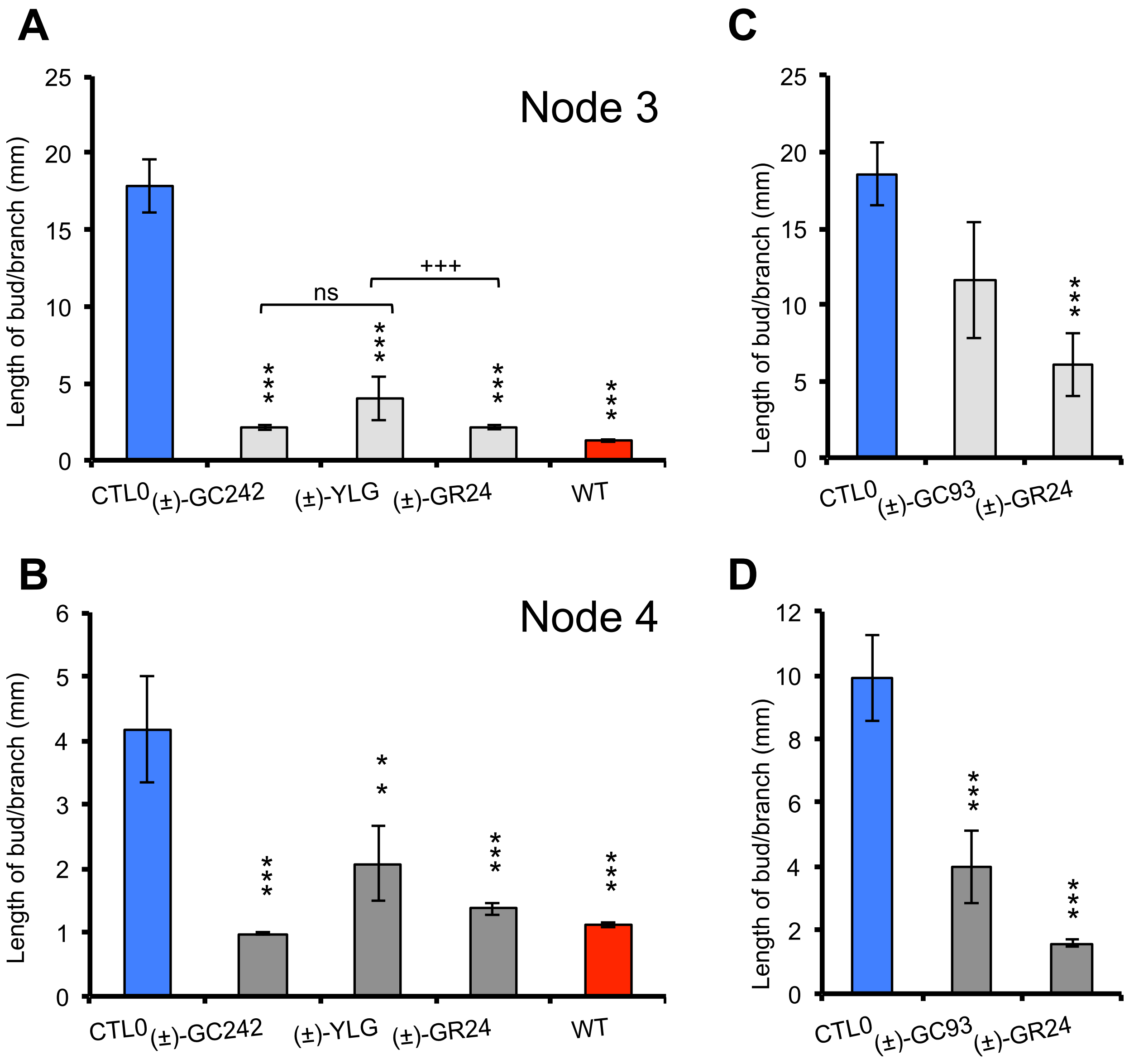

Supplemental Figure 2. Bud outgrowth inhibition activity assay results for SL analogs ((±)-GC242 and (±)-YLG (A,B); (±)-GC93 (C,D)) by vascular supply (3000 nM). [a] All the replicates are presented in Supplemental Table 2 with control 0. [b] These data were obtained from means ± SE (n = 12), 10 days after treatment. . * P < 0.05, ** P < 0.01, *** P < 0.001 indicate significant differences with the control treatment (0 nM) (Kruskal–Wallis rank sum test). CTL 0 = control 0. [+++] P < 0.001 indicates significant differences with the (±)-GC242 treatment (3000 nM) (Kruskal–Wallis rank sum test). ns = not significant.

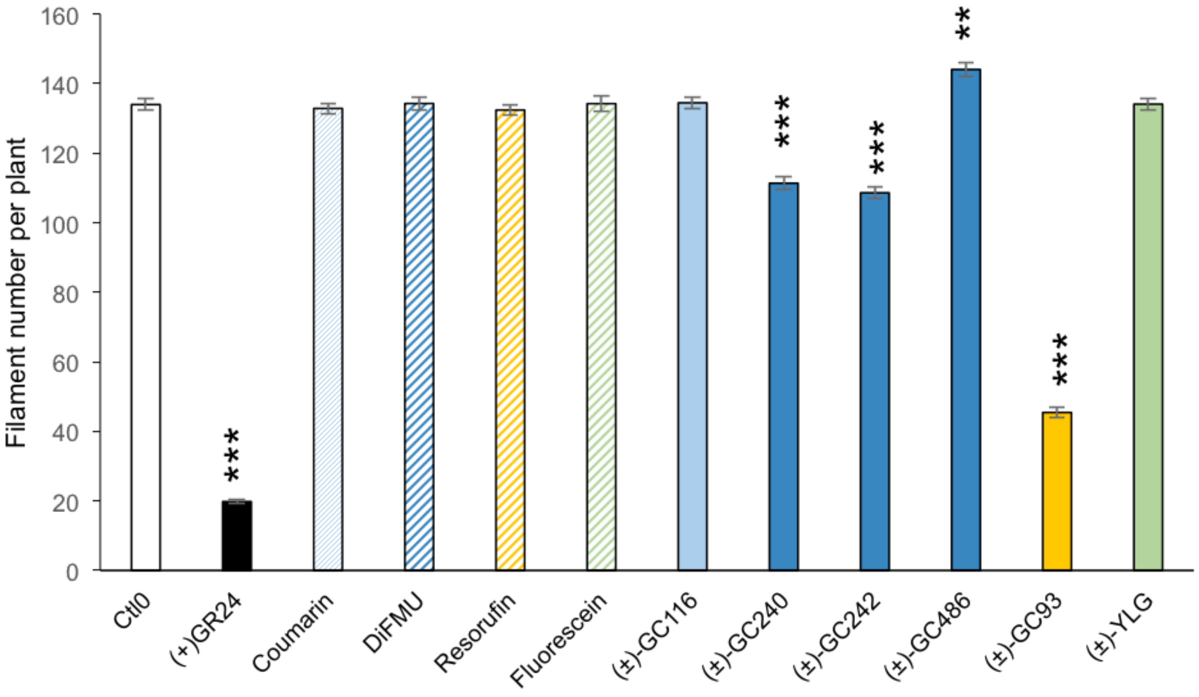

Supplemental Figure 3. Probe bioactivity tested on the moss *P. patens*. Bioactivity of fluorophores and profluorescent probes was assayed by counting the number of filaments grown in the dark following application, and compared to that of DMSO (control 0, white), and (+)-GR24 (in black). Each compound was used at 1 **µ**M. Fluorophore bars are hatched, while pro-fluorescent probe bars are filled. Data are mean ± SE of 24 *Ppccd8* mutant plants (n = 24) grown in 3 different 24-well plates. Significant differences between the control treatment (0 nM) and treated plants are based on ANOVA and Tukey Test as post hoc test; ** $P < 0.01$,*** $P < 0.001$. CTL0 = control 0.

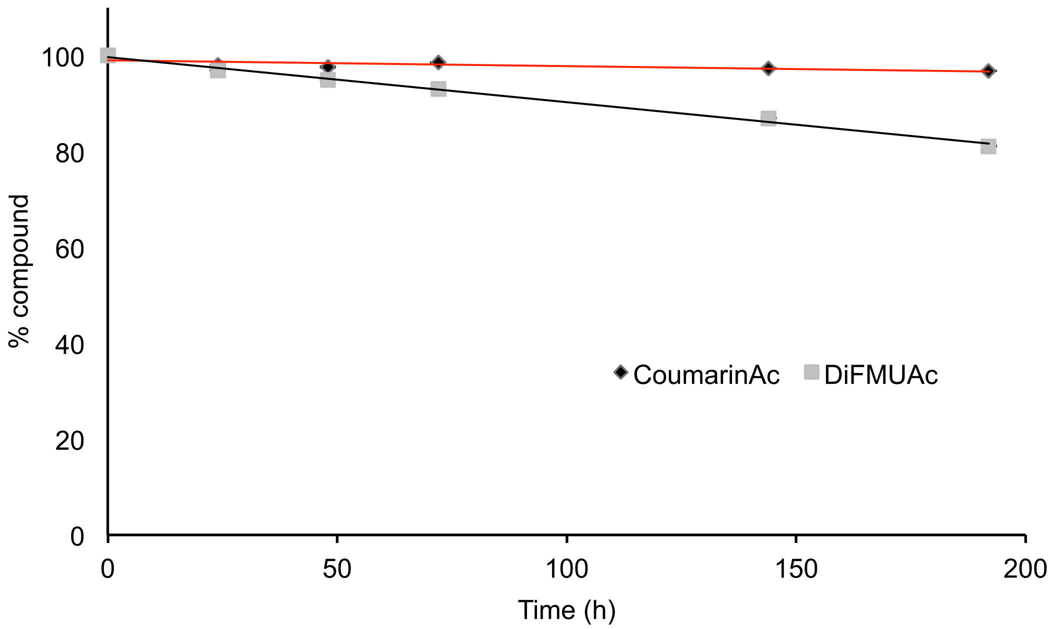

Supplemental Figure 4. Stability of CoumarinAc and DiFMUAc. Chemical hydrolysis of CoumarinAc and DiFMUAc in DMSO at 20 °C. Data are means ± SE (n = 3).

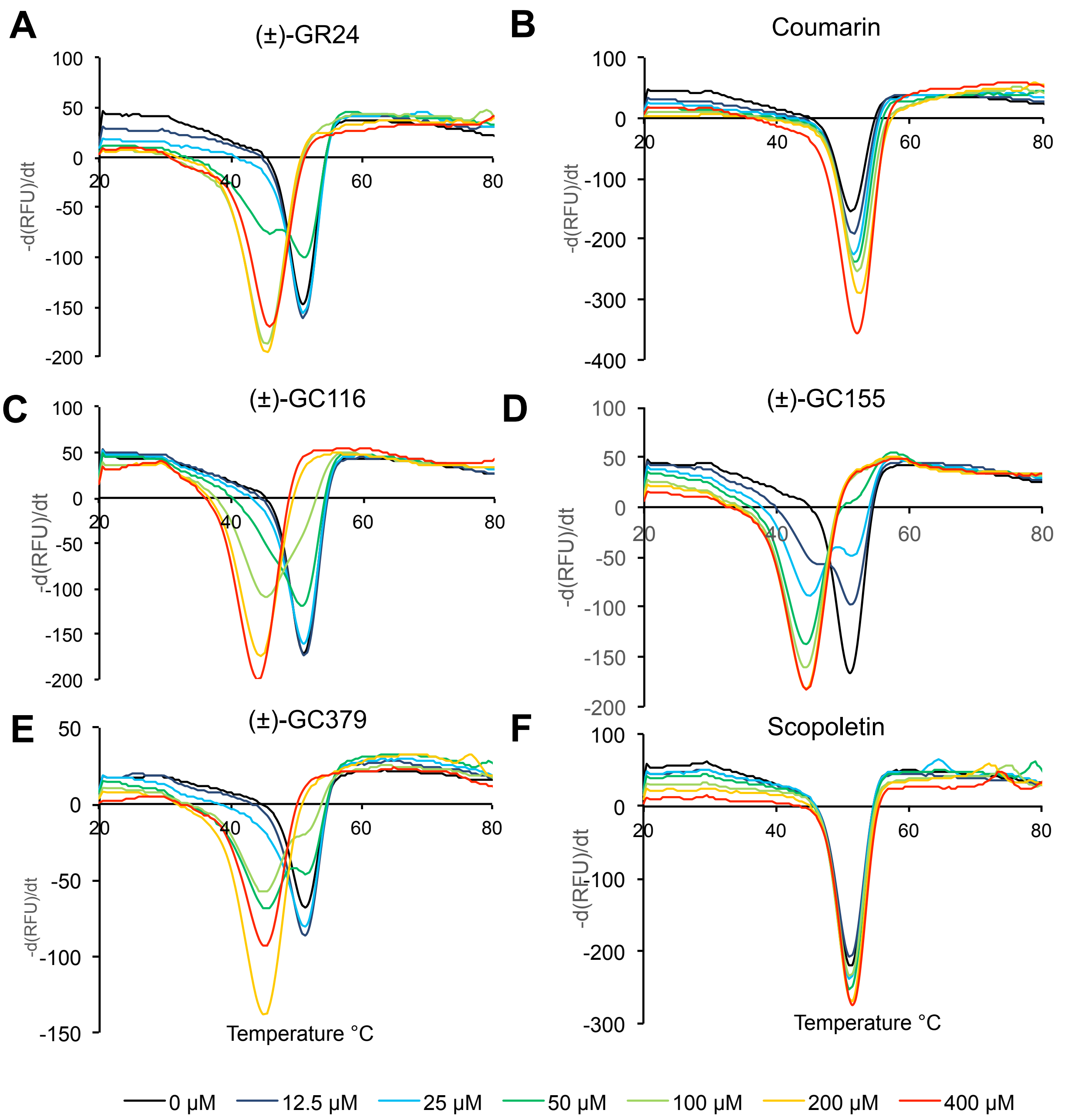

Supplemental Figure 5. DSF assay with SL profluorescent probes. The melting temperature curves of 10 µM RMS3 with (±)-GR24 (**A**), coumarin (**B**), (±)-GC116 (**C**), (±)-GC155 (**D**), (±)-GC379 (**E**) and scopoletin (**F**) at varying concentrations are shown as assessed by DSF. Each line represents
